# Covalent Attachment of Aggregation-Induced Emission Molecules to the Surface of Ultrasmall Gold Nanoparticles to Enhance Cell Penetration

**DOI:** 10.3390/molecules27061788

**Published:** 2022-03-09

**Authors:** Kai Klein, Matthias Hayduk, Sebastian Kollenda, Marco Schmiedtchen, Jens Voskuhl, Matthias Epple

**Affiliations:** 1 Inorganic Chemistry and Center for Nanointegration Duisburg-Essen (CeNIDE), University of Duisburg-Essen, Universitätsstr. 5-7, 45117 Essen, Germany; kai.klein@uni-due.de (K.K.); sebastian.kollenda@uni-due.de (S.K.); 2 Organic Chemistry and Center for Nanointegration Duisburg-Essen (CeNIDE), University of Duisburg-Essen, Universitätsstr. 5-7, 45117 Essen, Germany; matthias.hayduk@uni-due.de (M.H.); marco.schmiedtchen@stud.uni-due.de (M.S.)

**Keywords:** gold, nanoparticles, click chemistry, aggregation-induced emission, bioavailability

## Abstract

Three different alkyne-terminated aggregation-induced emission molecules based on a *para*-substituted di-thioether were attached to the surface of ultrasmall gold nanoparticles (2 nm) by copper-catalyzed azide–alkyne cycloaddition (click chemistry). They showed a strong fluorescence and were well water-dispersible, in contrast to the dissolved AIE molecules. The AIE-loaded nanoparticles were not cytotoxic and easily penetrated the membrane of HeLa cells, paving the way for an intracellular application of AIE molecules, e.g., for imaging.

## 1. Introduction

Ultrasmall metallic nanoparticles are versatile tools for imaging, catalysis, and drug delivery [1,2,3,4,5,6,7,8,9,10,11,12]. Chemically, they are at the borderline between metallic nanoparticles and atom-sharp metal clusters [4,13,14,15,16]. Due to their small size (about 2 nm), they easily penetrate cell walls [7,17,18] and, in favorable cases, also the nuclear membrane [19,20,21] and the blood–brain barrier [22,23]. Their synthesis is usually performed “bottom-up” by the reduction of metal salts with NaBH_4_, following the Brust–Schiffrin synthesis [24,25]. Recently, they have also been prepared “top-down” by pulsed laser ablation in the form of unprotected gold clusters [26]. We have reported previously how ultrasmall gold nanoparticles can be covalently surface-functionalized with dyes or protein binders via copper-catalyzed azide–alkyne cycloaddition (click chemistry) onto azide-terminated gold nanoparticles [19,22,23,27].

Here, we extend this concept to aggregation-induced emission molecules (AIE). The phenomenon that some compounds show emission when aggregated or bound to a specific target has been known for decades and has been used in numerous applications [28]. In 2001, this renaissance of AIE occurred when Tang et al. discovered that 1-methyl-1,2,3,4,5-pentaphenylsilole gives a bright emission in the aggregated and solid state, but not when it is molecularly dissolved [29]. This effect was labeled aggregation-induced emission and has attracted considerable attention in areas such as biomedicine and materials science [30,31]. However, the term AIE is somewhat misleading, because emission enhancement is not only observed when aggregation occurs but also when a single compound is constrained in its molecular rotation or vibration [32]. This fixation of motion leads to an inhibition of non-radiative decay, and hence emission is observed [33]. It is noteworthy that this very feature enables AIE molecules to act as a reporter for binding events, which makes them ideal candidates for imaging purposes.

In the last decade, attempts to prepare nanoparticles consisting of AIE compounds or an attachment of these luminophores to nanoparticles have been described. Qian et al. described tetraphenylethene (TPE)-derivatives encapsulated in a polymer matrix yielding nanoparticles for photodynamic therapy [34]. Liu et al. reported TPE-derived amphiphiles which were able to self-assemble into nanoparticles to deliver drugs such as doxorubicin into the cytosole [35]. Metal clusters were also found to have AIE features (luminescent metal nanoclusters with aggregation-induced emission). For instance, gold nanoclusters emitted over a broad range with remarkable quantum yields [36].

Here, we describe the covalent attachment of AIE emitters by Cu(I)-catalyzed azide–alkyne cycloaddition to the surface of ultrasmall gold nanoparticles and demonstrate their potential as imaging agents. Unlike larger gold nanoparticles (10 nm or more) [37,38], ultrasmall gold nanoparticles do not quench the emission of attached fluorophores [39], making them excellent carriers for AIE molecules.

## 2. Results

**Figure 1:**
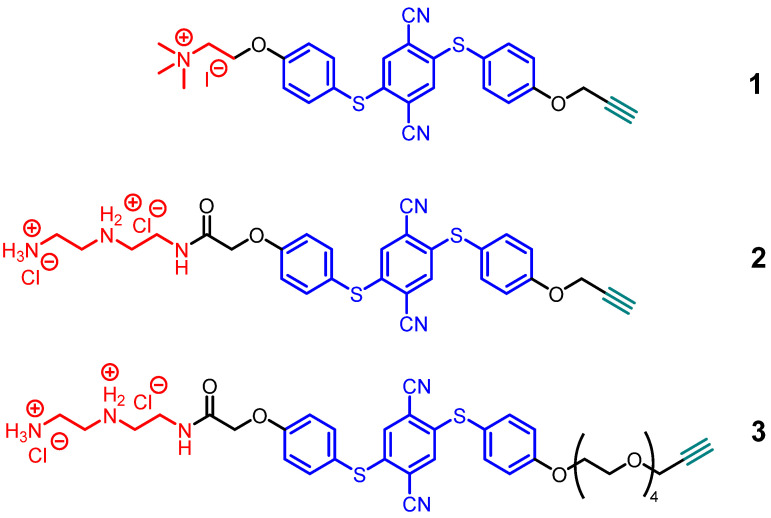
Molecular structures of the AIE ligands **1** to **3** that were covalently attached to ultrasmall gold nanoparticles.

Three AIE ligands with the same fluorophore, but with different polarity and hydrophilicity, were investigated (Figure 1). They were based on a known fluorophore [40] and functionalized with an alkyne group for covalent attachment to the nanoparticles. The photophysical properties of the AIE molecules were assessed by UV/Vis and fluorescence spectroscopy. To this end, the ligands were dissolved in an appropriate solvent and aggregated by the addition of a non-solvent. All ligands showed the expected UV/Vis absorption between 380 and 400 nm, which agrees with their yellow color in the aggregated (in THF–water or water–DMF mixtures) and in the solid state (see Supplementary Materials for details). Since the core motif is based on a *para*-substituted di-thioether for all compounds, the emission spectra agreed well with the free bisphenol described earlier [40]. The emission intensity of **2** and **3** increased drastically at high THF contents in water (99%) (Supporting Information) due to its amphiphilic character, which prevents an efficient aggregation in solvent mixtures with high water contents due to its solubility. For compound **1**, the amphiphilic character in combination with iodide as counter-ion led to a generally low emission, independent from its aggregation state, due to the heavy atom effect of iodide ions [41].

Compounds **2** and **3** were aggregated by the addition of THF to the aqueous solution (Supporting Information). It is noteworthy that the solid-state emission spectra matched the spectra in the aggregated state, because the aggregates can be regarded as dispersed solids.

**Figure 2:**
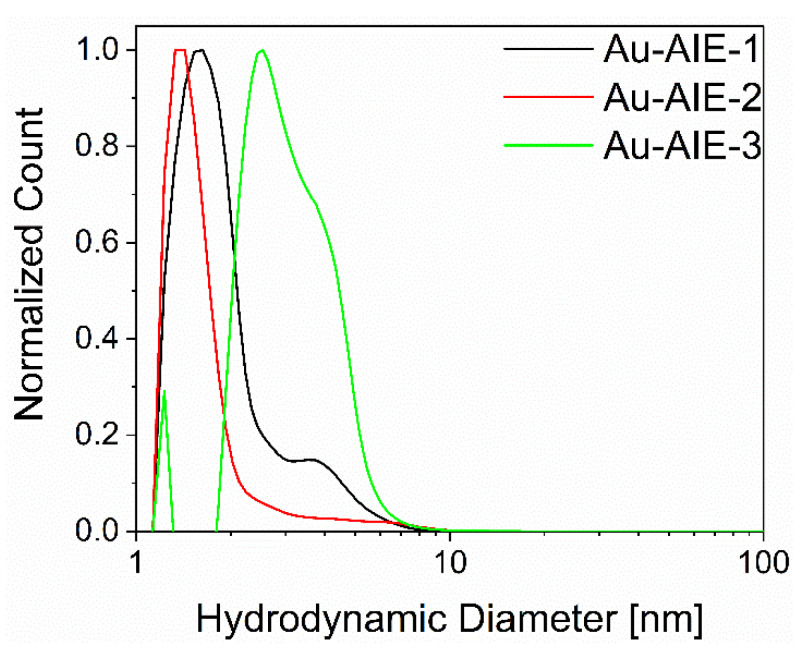
Normalized DCS number-weighted particle size distributions of **Au-AIE** nanoparticles, dispersed in water. The particles are well dispersed, with hydrodynamic diameters of 1.7 nm (**Au-AIE-1**), 1.4 nm (**Au-AIE-2**), and 3.2 nm (**Au-AIE-3**).

The AIE luminophores **1** to **3** were then clicked to the surface of azide-terminated glutathione-stabilized ultrasmall gold nanoparticles (2 nm) [39] to enhance their dispersibility in water and their ability to enter cells. Disc centrifugal sedimentation (DCS) gave the hydrodynamic particle diameter (Figure 2). The **Au-AIE-3** nanoparticles were bigger (3.2 nm) than the two other particles (1.4 and 1.7 nm) due to the hydrated (PEG)_4_ group that contributed to the hydrodynamic diameter. The gold nanoparticles before copper-catalyzed azide–alkyne click reaction (CuAAc) had a hydrodynamic diameter by DCS of 1.5 nm [39]. Note that DCS systematically underestimates the particle diameter due to the hydration shell [42].

^1^H-NMR spectroscopy was carried out to exclude the presence of residual impurities of unattached AIE fluorophores which would be detectable by very sharp signals instead of the broadened ones of particle-bound molecules [43,44]. The AIE-clicked nanoparticles were dispersed in D_2_O/DMSO-*d*_6_ (*v*:*v* = 1:2) because they tended to aggregate at the high concentrations necessary for NMR spectroscopy (10–15 g Au L^−1^). The spectra of all functionalized nanoparticles showed very distinct broad signals and no narrow NMR peaks between 7 and 7.5 ppm, indicating the aromatic parts of the intact AIE molecules.

UV/Vis spectroscopy gave the concentration of AIE molecules attached to the nanoparticle surface. By comparison with the concentration of nanoparticles in the dispersion (assessed from the gold concentration by atomic absorption spectroscopy (AAS) by assuming spherical gold nanoparticles with an average diameter of 2 nm [39]), the number of ligands attached to each nanoparticle was determined. Each nanoparticle carried between 50 and 80 AIE molecules, depending on the ligand. As expected, the PEGylated molecule **3** needed more space than the molecules **1** and **2**. The molecular footprint of 0.15 to 0.25 nm^2^ was low compared to other clicked dyes such as fluorescein (FAM; 1.48 nm^2^ [19,22] and 2.1 nm^2^ [39]), AlexaFluor647 (1.15 nm^2^ [39]), and Cy3 (2.5 nm^2^ [22]). In other words, the loading with AIE molecules was high and the packing was high compared to other dyes. Table 1 summarizes the major characterization data.

**Figure 3:**
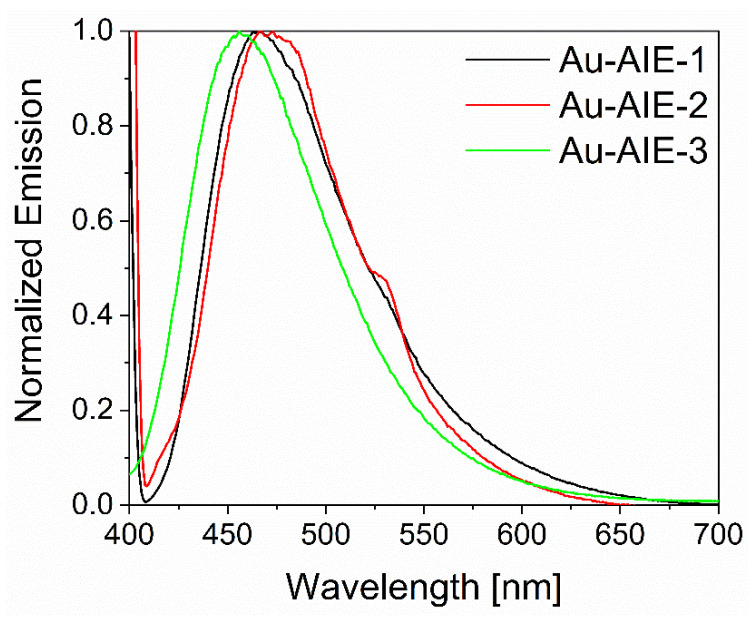
Normalized fluorescence emission spectra of **Au-AIE** nanoparticles, dispersed in water (excitation wavelength 365 nm). The emission peaks at and below 400 nm are due to scattering from the excitation source.

To ensure that the emission characteristics remained intact after attachment to the surface of ultrasmall gold nanoparticles, fluorescence spectroscopy was performed on water-dispersed **Au-AIE** nanoparticles (Figure 3). The excitation and emission wavelengths of the particle-bound fluorophores were the same as with the dissolved fluorophores (see Supplementary Materials).

In general, the fluorescence intensity depends on the solubility of the AIE fluorophore within the solvent. If the molecule rotates freely, non-radiative deactivation pathways are likely to occur, leading to a vanishing of potential emission. If the fluorophore is attached to a particle surface, three potential scenarios may occur:

(1) The luminophores are bound on a single site to the particle but the rotation of the luminophore remains intact, hence no emission is expected;
(2) The packing density of the organic shell on the surface is high enough to constrain the motion of ligands, leading to fluorescence emission;
(3) The ligand folds back to the surface, leading to a fixation of the luminophore, thereby inducing emission.

The fact that we observed a strong emission of the surface-bound AIE molecules indicates that the high surface density restricts the free rotation of the molecule (scenario two). However, a back-folding cannot be excluded (scenario three), but is unlikely due to the high surface loading and the small ligand footprint (Table 1).

**Figure 4:**
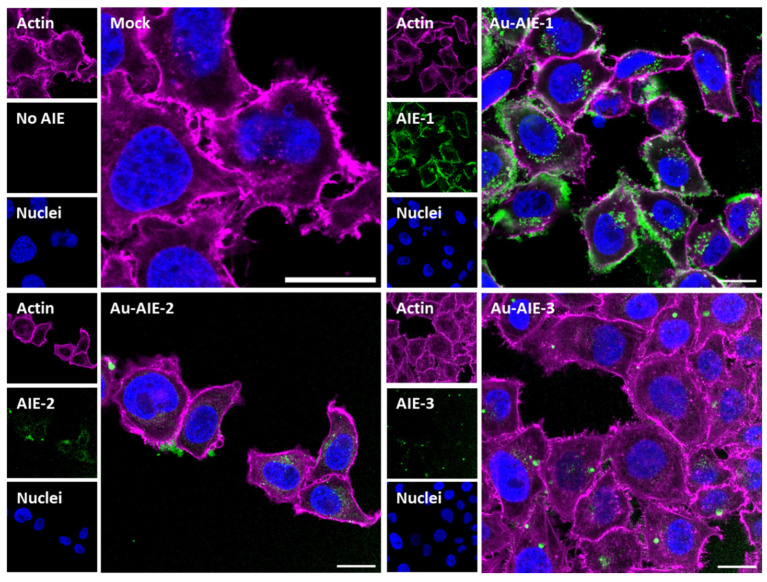
Confocal laser scanning images of HeLa Kyoto cells after incubation with **Au-AIE** nanoparticles, dispersed in DMEM. The cells were incubated with nanoparticles for 24 h, then washed, fixed, and stained. All scale bars are 20 µm.

The AIE molecules were well taken up by cells, in contrast to the dissolved molecules. Figure 4 shows confocal laser microscopic images of the uptake of AIE-loaded gold nanoparticles by HeLa Kyoto (H2B-mCherry) cells. All slices shown are taken from the focal plane in the middle of the whole cell volume (z-stacks).

All cells took up **Au-AIE** nanoparticles, although there were differences in the AIE signal intensity. **Au-AIE-1** was taken up most efficiently, possibly because it was the smallest molecule. The nanoparticle distribution inside the cells mostly resembled a vesicular enclosure but no free distribution throughout the cytoplasm. The dissolved AIE molecules alone were not taken up by cells but were adsorbed only on the cell wall (data not shown).

**Figure 5:**
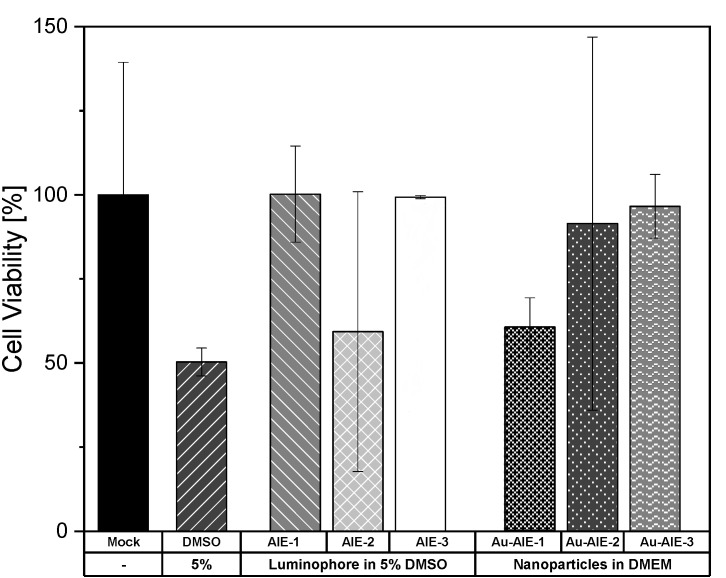
MTT cell viability assay with HeLa cells, incubated with water-dispersed **Au-AIE** gold nanoparticles (**Au-AIE-1**, **Au-AIE-2**, **Au-AIE-3**) and with dissolved AIE luminophores (in 5% DMSO). Data represent the mean of three individual experiments with the standard deviation.

Finally, the cytotoxicity of the dissolved ligands and of the dispersed **Au-AIE** nanoparticles was assessed (Figure 5). HeLa Kyoto (H2B-mCherry) cells were incubated with free AIE luminophores (dissolved in 5/95 vol% DMSO/DMEM). The overall viability of HeLa cells incubated with AIE luminophores ranged between 60 and 100%. Considering the error bars and the DMSO control group, this indicates that there are no cytotoxic effects coming from the luminophores themselves, but that the 5% DMSO content induced cell death. In comparison, the viability of HeLa cells incubated with nanoparticles dispersed in water was in the range of 60 to 97%, showing only a moderate impact on cell viability for **Au-AIE-1**. Altogether, the **Au-AIE** particles and the AIE molecules showed only a low cytotoxicity.

## 3. Materials and Methods

### 3.1. Chemicals

2-(4,5-dimethylthiazol-2-yl)-3,5-diphenyl-2*H*-tetrazol-3-ium bromide (MTT) was obtained from Thermo Fisher Scientific. Dimethyl sulfoxide (DMSO, ≥99.5%) was obtained from Carl Roth. Propargyl bromide, 4-mercaptophenol, potassium carbonate, 2-chloro-*N*,*N*-dimethylethylamine hydrochloride, methyl iodide, tert-butyl bromoacetate, trifluoroacetic acid, 1-ethyl-3-(3-dimethylaminopropyl)carbodiimide, 1-hydroxybenzotriazole, *N*-methylmorpholine, acetyl chloride, tetraethylene glycol, and p-toluenesulfonylchloride were obtained from TCI chemicals. 1,4-bis-boc-1,4,7-triazaheptane was obtained from Iris biotech. All reagents were used as delivered without further purification.

Ultrapure water was prepared with a Purelab ultra instrument from ELGA and used for all syntheses involving nanoparticles. All chemicals were used without further purification. Prior to all syntheses involving gold nanoparticles, the glassware was cleaned once with boiling aqua regia and twice with boiling ultrapure water.

### 3.2. Nanoparticle Synthesis

Glutathione-stabilized gold nanoparticles with a diameter of 2 nm were prepared and azide-terminated as reported earlier (see ref. [39] for a full characterization of gold-glutathione and gold-azide nanoparticles). Each gold nanoparticle carried 118 azide groups for clicking, as determined by NMR spectroscopy [39]. Clicking of alkyne-terminated fluorophores was performed by copper-catalyzed azide–alkyne cycloaddition (CuAAC) [45]. One hundred and eighteen equivalents (1 eq. to N_3_; 5 µmol AIE dye, i.e., 3.14 mg **1**, 3.14 mg **2**, 4.02 mg **3**, respectively) of AIE molecules to nanoparticles were dissolved in 2.8 mL DMSO, respectively. The AuN_3_ nanoparticle dispersion (2 mg Au, 41 nmol NPs in 0.5 mL water) was added to the AIE solution. CuSO_4_ (14.25 eq. to nanoparticles), THPTA (71.25 eq. to nanoparticles), and aminoguanidinium hydrogen carbonate (71.25 eq. to nanoparticles) were dissolved in 0.1 mL water at 60 °C and added to the solution after cooling to ambient temperature; then, sodium ascorbate (17 eq. to nanoparticles in 0.1 mL water) was added. The suspension was stirred for 48 h at room temperature to ensure an efficient clicking reaction. Afterwards, the suspension was diluted with 56 mL water to <5% DMSO, and then the nanoparticles were isolated by 3 kDa Amicon © spin filtration (Amicon, Millipore, Burlington, MA, USA) (4000 rpm, 2500× *g*) and multiply washed with water to remove residual copper, excess clicking reagents, and unbound ligands. 

The number of AIE molecules that had been clicked to the surface of each nanoparticle was determined from the ratio of the concentrations of nanoparticles and AIE molecules in a given dispersion of **Au-AIE** nanoparticles [39]. The nanoparticle concentration *c*_NP_ was calculated from the gold concentration *c*_Au_ as:

(1) cNP=cAuNA×ρ×43πr3

The diameter of the particles was assumed as 2 nm, as determined earlier by HRTEM [39]; ρ is the density of gold (19.32 g cm^−3^), and N_A_ is Avogadro’s constant. The AIE concentration was determined by UV/Vis spectroscopy of the dispersions in DMSO:water (2:1), followed by quantitative analysis with previously prepared calibration lines for the dissolved AIE ligands. All nanoparticle dispersions were adjusted to a gold concentration of 30 mg L^−1^. The AIE-labelled nanoparticles are denoted as **Au-AIE** in general and as **Au-AIE-1**, **Au-AIE-2**, and **Au-AIE-3** for the individual particles.

### 3.3. AIE Ligand Synthesis and Characterization 

The synthesis of the three AIE ligands started from dimethyldibromobenzene, which was converted in a three-step reaction into 2,5-dibromoterepthalonitrile **A** [46,47]. This nitrile was reacted with 4-mercaptophenol, yielding **B** [48], treated with propargyl bromide, and reacted once more with 4-mercaptophenol, leading to **D**. The reaction with 2-chloro-*N,N*-dimethylethylamine hydrochloride and the final methylation with methyl iodide gave **1** in good yield. The reaction of tert-butyl bromoacetate with **D**, followed by treatment with trifluoroacetic acid, led to compound **G**. Molecule **2** was obtained by coupling with 1,4-bis-boc-1,4,7-triazaheptane, followed by deprotection with acetylchloride in good yield. The PEGylated molecule **3** was synthesized differently. Dibromoterepthalonitrile was reacted with 4-mercaptophenol, giving bisphenol **O** [40], followed by substitution with tert-butyl bromoacetate, yielding **K**. Coupling of the tetraethyleneglycol derivative **J** [49] to the free phenol and acidic deprotection of the tert-butyl protection group gave **M**. Peptide-coupling with 1,4-bis-boc-1,4,7-triazaheptane, followed by deprotection with acetylchloride, gave **3**. 

All molecules were fully characterized by ^1^H and ^13^C-NMR, HR-ESI-MS, and IR spectroscopy (for full details of synthesis and characterization see Supplementary Materials). 

### 3.4. Cells and Cell Culture

The human cervix carcinoma cell line HeLa Kyoto H2B-mCherry was a gift from Prof. Hemmo Meyer (University of Duisburg-Essen, Faculty of Biology). The cells were cultivated at 37 °C and 5% CO_2_ in Gibco^TM^ Dulbecco’s modified Eagle’s medium (DMEM, Thermo Fisher Scientific, Waltham, MA, USA) supplemented with 10% fetal bovine serum (FBS, Thermo Fisher Scientific), 100 U mL^−1^ Gibco^TM^ penicillin–streptomycin (Thermo Fisher Scientific), 1 mM Gibco^TM^ sodium pyruvate (Thermo Fisher Scientific), and 1 mM Gibco^TM^ GlutaMAX. Cells were passaged at 70–90% confluency or every two to three days by trypsinization with 0.05% Gibco^TM^ Trypsin–EDTA (Thermo Fisher Scientific). The cells were washed two to three times with Gibco^TM^ Dulbecco’s buffered saline (DPBS, Thermo Fisher Scientific) between the individual steps of each experiment.

### 3.5. Cytotoxicity by the MTT Test 

The cell viability was determined by an MTT assay. First, HeLa cells were seeded at a density of 50,000 cells per well in a 24-well plate and incubated with 0.5 mL DMEM overnight at 37 °C in 5% CO_2_ atmosphere. Next, the cells were incubated with 500 µL of a 3.06 µM solution of AIE molecules in 5/95 vol% DMSO/DMEM. Five hundred µL of AIE-clicked ultrasmall gold nanoparticles (12.5 µg gold), dispersed in DMEM, were applied per well (25 µg mL^−1^ gold). Control groups were cells treated with 500 µL of a 5/95 vol% DMSO/DMEM mixture and cells cultivated in medium alone (viability 100%). After the incubation for 24 h, the cells were washed three times with DPBS to remove dissolved and weakly adhering AIE luminophores and nanoparticles, respectively. 

For the staining solution, 5 mg MTT were dissolved in 1 mL PBS and then diluted with 4 mL DMEM to a final concentration of 1 mg mL^−1^. Into each well was pipetted 0.3 mL of the staining solution, and the cells were incubated for 1 h. Next, the solution was replaced with 0.3 mL DMSO and incubated for another 30 min. The dissolved formazan was quantitatively determined in a 96-well plate with a Multiscan plate reader (Thermo Fisher Scientific GmbH) at 570 nm.

### 3.6. Nanoparticle Uptake by Cells

Uptake experiments by HeLa Kyoto H2B-mCherry cells were carried out in an 8-well chamber polymer slide surface modified with ibiTreat for tissue culture applications (µ-Slide 8-well, ibidi). Twenty thousand cells were seeded per well and incubated with 0.2 mL DMEM overnight at 37 °C in 5% CO_2_ atmosphere. Similar to the viability tests, the cells were incubated with 200 µL of a 3.06 µM solution of AIE molecules in 5/95 vol% DMSO/DMEM. Two hundred µL of AIE-clicked ultrasmall gold nanoparticles (5 µg gold), dispersed in DMEM, were applied per well (25 µg Au mL^−1^). Control groups were cells treated with 200 µL of a 5/95 vol% DMSO/DMEM mixture and cells cultivated in medium alone. After incubation for 24 h, the cells were washed three times with DPBS, and fixed with 4 vol% formaldehyde solution according to standard protocols. For post-fixation, the actin skeleton of the cells was stained with AlexaFluor^TM^ 647 Phalloidin. Nuclei were recognizable without staining by the red fluorescence of the mCherry-labelled histone-binding protein H2B.

### 3.7. Analytical Methods

The concentration of gold in the particle dispersion was measured by atomic absorption spectroscopy (AAS) in an ICE 3000 spectrometer (Thermo Fischer Scientific) after chemical dissolution (oxidation) of the gold nanoparticles *in aqua regia*. The concentration of gold was then used to determine the concentration of nanoparticles. UV/Vis measurements on dissolved and nanoparticle-conjugated AIE fluorophores were performed with a Thermo Scientific Genesys 50 instrument. For the calibration rows, the fluorophores were dissolved in a mixture of DMSO and water (9:1 = *v*:*v*) at different concentrations. Fluorescence spectroscopy was performed with an Agilent Technologies Cary Eclipse Spectrophotometer. The nanoparticles were dispersed in water and measured in a 600 µL quartz glass cuvette. Differential centrifugal sedimentation (DCS) was performed with a DC24000 UHR instrument (CPS Instruments) with a sucrose gradient, capped with 0.5 mL dodecane. The calibration was performed with dispersed PVC standard particles (diameter 483 nm). ^1^H-NMR spectroscopy was performed with an AV Neo 400 instrument (Bruker, Rheinstetten, Germany) at 400 MHz. Nanoparticles with attached fluorophores were lyophilized and then redispersed in a mixture of DMSO-d_6_ and D_2_O (*v*:*v* = 9:1). NMR on ultrasmall particles is a reliable method to ensure that the ligands are attached to the particle, although the NMR signals are considerably broadened due to the vicinity of the metallic particle [43,44,50,51]. Multiple focal plane (z-stacks, interval 0.5 µm) confocal laser scanning microscopy (CLSM) on HeLa cells was performed with a TCS SP8X Falcon instrument (Leica Microsystems) with a 63×/1.2 water immersion objective. The excitation wavelength for AIE luminophores was 405 nm (emission: **1**, **2**, **3**; 510 to 540 nm), 561 nm for mCherry (emission: 580 to 600 nm), and 633 nm for AlexaFluor^TM^ 647 Phalloidin (emission: 640 to 700 nm).

## 4. Conclusions

AIE molecules can be covalently attached to the surface of ultrasmall gold nanoparticles with high efficiency (50 to 80 AIE molecules per 2 nm gold nanoparticle). Such particles are easily water-dispersible and show a high degree of fluorescence, probably due to the high surface density of the AIE molecules, which restricts their intramolecular motion. The fact that the nanoparticles are well taken up by cells, in contrast to the dissolved AIE molecules, underscores the potential of this approach to enhance the bioavailability of AIE molecules, e.g., for bioimaging.

## Figures and Tables

**Table 1:** Analytical data of AIE luminophores, clicked to gold nanoparticles at *c*(NP) = 617 nM (30 mg L^−1^ Au). To compute the number of AIE molecules on each particle, the diameter of the solid core was set to 2 nm, as known from HRTEM data [39]. The clicking efficiency was defined as the ratio of the number of clicked AIE ligands to the number of available azide groups (118) on each particle [39]. Note that the hydrodynamic diameter by DCS also includes the ligand shell.

	Au-AIE-1	Au-AIE-2	Au-AIE-3
Hydrodynamic particle diameter by DCS (nm)	1.7 ± 0.4	1.4 ± 0.2	3.2 ± 1.0
*c*(AIE) (µM)	44	52	32
*N*(AIE) (per nanoparticle)	71 ± 14	84 ± 16	51 ± 10
Clicking efficiency (%)	60	71	43
Molecular footprint per AIE molecule (nm^2^)	0.18	0.15	0.25

## Data Availability

Data is contained within the article or Supplementary Materials.

## Supplementary Materials

The following supporting information can be downloaded at: https://www.mdpi.com/article/10.3390/molecules27061788/s1, Details on synthesis and characterization of the AIE compounds.

## Sample Availability

Samples of the compounds are not available from the authors.
