# Supplementary material for: Covalent Attachment of Aggregation-Induced Emission Molecules to the Surface of Ultrasmall Gold Nanoparticles to Enhance Cell Penetration
Source: Molecules. 2022 Mar 9;27(6):1788. doi: 10.3390/molecules27061788 (PMC8949416; doi:10.3390/molecules27061788)
Supplement: Supplementary file 1 [file molecules-27-01788-s001.zip › molecules-1624537-supplementary.pdf]

molecules

Article

Covalent Attachment of Aggregation-Induced Emission Molecules to the Surface of Ultrasmall Gold Nanoparticles to Enhance Cell Penetration

Kai Klein [1], Matthias Hayduk [2], Sebastian Kollenda [1], Marco Schmiedtchen [2], Jens Voskuhl [2,*] and Matthias Epple [1,*]

[1] Inorganic Chemistry and Center for Nanointegration Duisburg-Essen (CeNIDE), University of Duisburg-Essen, Universitätsstr. 5-7, 45117 Essen, Germany; kai.klein@uni-due.de (K.K); sebastian.kollenda@uni-due.de (S.K.)

[2] Organic Chemistry and Center for Nanointegration Duisburg-Essen (CeNIDE), University of Duisburg-Essen, Universitätsstr. 5-7, 45117 Essen, Germany; matthias.hayduk@uni-due.de (M.H.); marco.schmiedtchen@stud.uni-due.de (M.S.)

* Correspondence: jens.voskuhl@uni-due.de (J.V.); matthias.epple@uni-due.de (M.E.)

1. General information and instrumentation

The chemicals were purchased from Sigma Aldrich, Iris Biotech, Carl Roth or TCI Chemicals, and were used without further purification. The reactions were carried out using dried solvents and under inert gas conditions (argon atmosphere). The reactions were monitored by thin-layer chromatography (TLC) on 0.2 mm Macherey-Nagel ALUGRAM precoated silica gel aluminium sheets. The spots were visualized by an UV-handlamp (λ = 254 and 365 nm) or with a basic $KMnO_4$ solution. For silica gel column chromatography silica gel 60 (0.063-0.2 mm, Merck) was used. Potassium carbonate was dried at 90 °C and freshly ground before use. DMF was freshly distilled and stored over molecular sieves 0.4 nm. The water was removed by using a freeze-dryer Alpha 1-4 LD plus from the company Martin Christ Gefriertrocknungsanlagen GmbH. A Bruker DMX 300 spectrometer, a DRX 400 spectrometer or a DMX 600 spectrometer were used to record ^{13}C and ^{1}H-NMR spectra at room temperature. The signals are referenced relative to the residual solvent signals (d_6-DMSO: δ = 2.50 ppm (^{1}H), δ = 39.52 ppm (^{13}C)). All coupling constants (*J*) are reported in Hertz (Hz). HR-ESI-MS spectra were recorded on a Bruker maXis 4G UHR-TOF or on a LTQ Orbitrap LTQ XL (Thermo-Fisher Scientific, Bremen). IR spectra were monitored using an IRTracer-100 (Shimadzu Corporation). Emission and excitation spectra were measured on a RF-6000 (Shimadzu). Emission spectra were divided by the peak maximum value within a measurement series yielding relative intensities. Solid samples were measured using a solid sample holder and quartz glass slides. A Jasco V-550 (JACA LTD) was used to measure UV-Vis spectra. Photographs were recorded on a Canon EOS 1100D.

2. Synthetic routes and procedures

The synthesis of (**A**)[1,2], (**B**)[3] and (**O**)[5] have been reported in literature. The analytical data obtained were in agreement with those reported in literature.

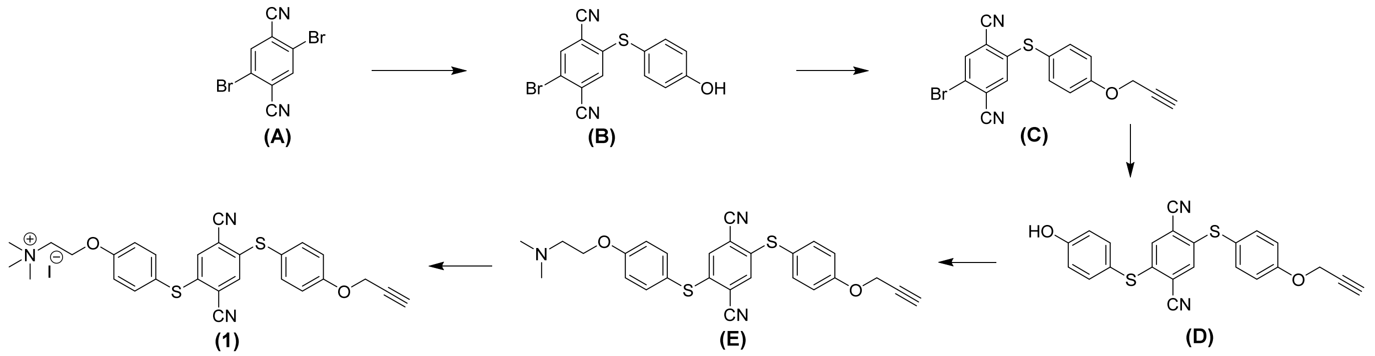

Scheme S1: Synthetic route to the clickable compound (**1**).

2-bromo-5-((4-(prop-2-yn-1-yloxy)phenyl)thio)terephthalonitrile (C)

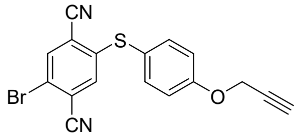

Compound **(B)** (1.50 g, 4.53 mmol) and potassium carbonate (1.87 g, 13.59 mmol) were added under argon to a 100 mL round bottom flask followed by the addition of dry DMF (20 mL). The suspension was stirred at 45 °C for 10 minutes. Then propargyl bromide (1.08 g, 9.06 mmol) was added via a syringe. The suspension was stirred at 45 °C for 3 h. The reaction was quenched carefully with 4 M HCl (100 mL) and the product precipitated as a white-brownish solid. This solid was separated by filtration and washed with distilled water. The product was dried *in vacuo* and obtained as a slightly brownish solid. (Yield 1.64 mg, 98%). Molecular mass: 369.24 g/mol, Molecular formula: $C_{17}H_9BrN_2OS$, ^{1}H-NMR (300 MHz, DMSO-d_6) δ = 8.51 (*s*, 1H), 7.57 (*d*, *J* = 8.9 Hz, 2H), 7.38 (*s*, 1H), 7.15 (*d*, *J* = 8.9 Hz, 2H), 4.89 (*d*, *J* = 2.4 Hz, 2H), 3.63 (*t*, *J* = 2.4 Hz, 1H) ppm. ^{13}C-NMR (75 MHz, DMSO-d_6) δ = 158.84, 143.47, 137.57, 136.28, 133.11, 121.39, 119.49, 119.34, 116.85, 115.90, 115.34, 114.47, 78.71, 78.67, 55.72 ppm. IR (ATR, $\tilde{\nu}$/cm^{-1}): 3279, 3079, 3007, 2230, 2128, 1591, 1574, 1491, 1458, 1408, 1375, 1337, 1290, 1263, 1240, 1173, 1152, 1109, 1098, 1086, 1022, 1009, 964, 912, 826, 802, 721, 698, 689, 662, 625. HR-APCI (toluene): m/z = 367.96106. (calcd. 367.96135 for $[C_{17}H_9BrN_2OS]^+$).

2-((4-hydroxyphenyl)thio)-5-((4-(prop-2-yn-1-yloxy)phenyl)thio)terephthalonitrile (D)

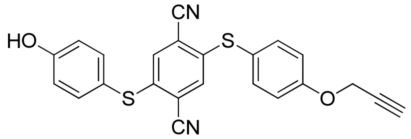

Compound **(C)** (1.00 g, 2.71 mmol), 4-mercaptophenol (308 mg, 2.44 mmol) and potassium carbonate (2.24 g, 16.20 mmol) were added under argon to a 100 mL round bottom flask followed by the addition of dry DMF (20 mL). The suspension was stirred at 45 °C for 6 h. The reaction was quenched carefully with 4 M HCl (100 mL) and the product precipitated as a yellow solid. This solid was separated by filtration and washed with distilled water. The product was purified using standard column chromatography (SiO_2) with cyclohexane and ethyl acetate (v:v = 6:1 → pure ethyl acetate) as solvents. The product was dried *in vacuo* and obtained as a yellow solid. (Yield 802 mg, 79%). Molecular mass: 414.50 g/mol, Molecular formula: $C_{23}H_{14}N_2O_2S_2$, ^{1}H-NMR (300 MHz, DMSO-d_6) δ = 10.16 (*s*, 1H), 7.53 (*d*, *J* = 8.7 Hz, 1H), 7.46 (*s*, *J* = 3.4 Hz, 1H), 7.42 (*d*, *J* = 8.6 Hz, 1H), 7.25 (*s*, 1H), 7.11 (*d*, *J* = 8.8 Hz, 1H), 6.91 (*d*, *J* = 8.6 Hz, 1H), 4.87 (*d*, *J* = 2.4 Hz, 1H), 3.62 (*t*, *J* = 2.3 Hz, 1H) ppm. ^{13}C-NMR (75 MHz, DMSO-d_6) δ = 159.55, 158.54, 142.42, 139.72, 136.78, 135.74, 133.60, 132.08, 120.62, 117.42, 116.69, 116.30, 115.76, 115.30, 115.16, 114.34, 78.75, 78.63, 55.68 ppm. IR (ATR, $\tilde{\nu}$/cm^{-1}): 3362, 3285, 3079, 2926, 2857, 2658, 2380, 2347, 2324, 2239, 2124, 1993, 1896, 1775, 1605, 1584, 1572, 1535, 1491, 1447, 1437, 1408, 1368, 1346, 1304, 1281, 1261, 1215, 1177, 1167, 1103, 1096, 1022, 1013, 939, 930, 899, 880, 831, 818, 810, 795, 723, 706, 683, 656, 646, 638, 623. HR-ESI-pos (MeOH): m/z = 437.0385. (calcd. 437.0389 for $[C_{23}H_{14}N_2O_2S_2Na]^+$).

2-((4-(2-(dimethylamino)ethoxy)phenyl)thio)-5-((4-(prop-2-yn-1-yloxy)phenyl)thio)terephthalonitrile (E)

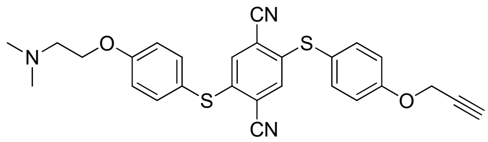

Compound **(D)** (200 mg, 0.48 mmol), 2-chloro-*N*,*N*-dimethylethylamine hydrochloride (62 mg, 0.58 mmol) and potassium carbonate (270 mg, 1.95 mmol) were added under argon to a 50 mL round bottom flask followed by the addition of dry DMF (10 mL). The suspension was stirred at 45 °C for 48 h. The reaction was quenched with water (100 mL) and the product precipitated as a yellow solid. This solid was separated by filtration and washed with distilled water. The product was purified using standard column chromatography (SiO_2) with dichloromethane and methanol (v:v = 95:5) as solvents. The product was dried *in vacuo* and obtained as a

yellow solid. (Yield 155 mg, 67%). Molecular mass: 485.62 g/mol, Molecular formula: $C_{27}H_{23}N_3O_2S_2$, ^{1}H-NMR (300 MHz, DMSO-d_6) δ = 7.56 – 7.48 (*m*, 4H), 7.44 (*s*, 1H), 7.38 (*s*, 1H), 7.15 – 7.06 (*m*, 4H), 4.88 (*d*, *J* = 2.4 Hz, 2H), 4.11 (*t*, *J* = 5.8 Hz, 2H), 3.62 (*t*, *J* = 2.3 Hz, 1H), 2.66 (*t*, *J* = 5.7 Hz, 2H), 2.23 (*s*, 6H) ppm. ^{13}C-NMR (75 MHz, DMSO-d_6) δ = 160.03, 158.60, 141.31, 140.43, 136.21, 135.85, 133.42, 132.93, 131.63, 119.04, 116.72, 116.45, 115.56, 115.24, 115.19, 115.12, 78.74, 78.64, 65.98, 57.46, 55.69, 45.41 ppm. IR (ATR, $\tilde{\nu}$/cm^{-1}): 3281, 2776, 2224, 2128, 1589, 1570, 1489, 1449, 1408, 1373, 1344, 1289, 1240, 1173, 1103, 1018, 963, 920, 882, 831, 808, 723, 708, 644. HR-ESI-pos (MeOH): m/z = 486.1306. (calcd. 486.1304 for $[C_{27}H_{23}N_3O_2S_2 + H]^+$).

2-(4-((2,5-dicyano-4-((4-(prop-2-yn-1-yloxy)phenyl)thio)phenyl)thio)phenoxy)-N,N,N-trimethylethanaminium iodide (1)

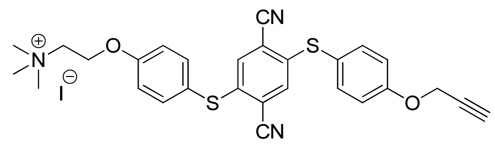

Compound **(E)** (60 mg, 0.12 mmol) was added to a 25 mL round bottom flask followed by the addition of DCM (8 mL). Then methyl iodide (1.0 mL, 16.06 mmol) was added via syringe injection. The solution was stirred at 45 °C for 24 h. Afterwards the excess of methyl iodide was distilled off and quenched with an aqueous ammonia solution. The residue obtained was dried *in vacuo* and the product was obtained as a yellow solid. (Yield 74 mg, 95%). Molecular mass: 627.56 g/mol, Molecular formula: $C_{28}H_{26}IN_3O_2S_2$, ^{1}H-NMR (600 MHz, DMSO-d_6) δ = 7.59 (*d*, *J* = 8.7 Hz, 2H), 7.55 (*d*, *J* = 8.7 Hz, 2H), 7.47 (*s*, 1H), 7.43 (*s*, 1H), 7.17 (*d*, *J* = 8.8 Hz, 2H), 7.14 (*d*, *J* = 8.8 Hz, 2H), 4.90 (*d*, *J* = 2.2 Hz, 2H), 4.54 (*s*, 2H), 3.84 – 3.81 (*m*, 2H), 3.64 (*t*, *J* = 2.1 Hz, 1H), 3.20 (*s*, 9H) ppm. ^{13}C-NMR (151 MHz, DMSO-d_6) δ = 158.86, 158.64, 140.85, 140.76, 136.08, 135.95, 133.41, 133.13, 120.31, 120.24, 116.75, 116.66, 115.44, 115.28, 115.21, 78.76, 78.70, 64.03, 61.89, 55.71, 53.12 ppm. (2 C's of the central aromatic unit overlap). IR (ATR, $\tilde{\nu}$/cm^{-1}): 3281, 3003, 2226, 2122, 1587, 1572, 1489, 1449, 1406, 1370, 1344, 1290, 1246, 1221, 1175, 1103, 1090, 1063, 1015, 955, 930, 883, 829, 808, 723, 708, 675, 646. HR-ESI-pos (MeOH): m/z = 500.1457. (calcd. 500.1461 for $[C_{28}H_{26}IN_3O_2S_2]^+$).

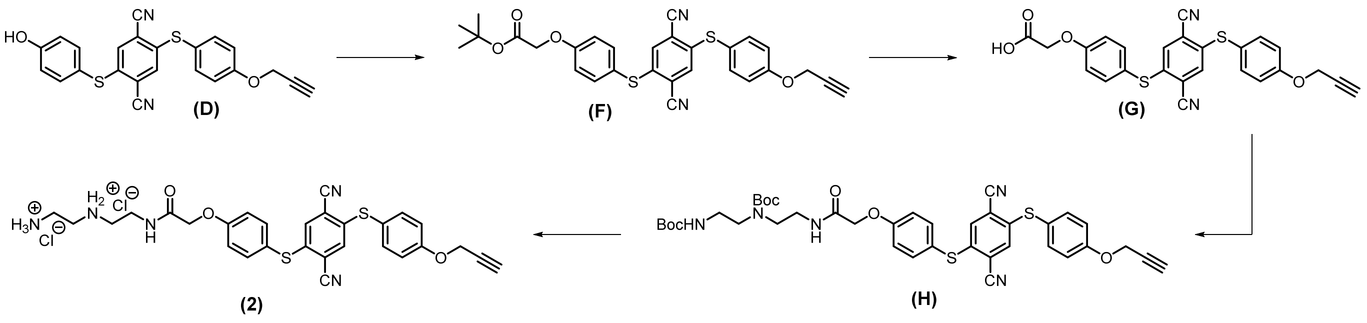

Scheme S2: Synthetic route to the clickable compound (**2**).

***tert*-butyl 2-(4-((2,5-dicyano-4-((4-(prop-2-yn-1-yloxy)phenyl)thio)phenyl)thio)phenoxy)acetate (F)**

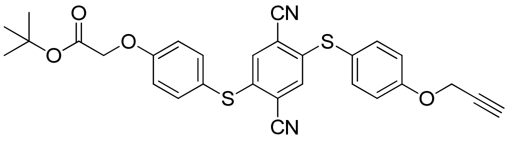

Compound **(D)** (145 mg, 0.35 mmol) and potassium carbonate (145 mg, 1.05 mmol) were added under argon to a 50 mL round bottom flask followed by the addition of dry DMF (10 mL). The solution was stirred at 45 °C for 10 minutes. Then *tert*-butyl bromoacetate (0.2 mL, 1.40 mmol) was injected dropwise via a syringe. The solution was stirred at 45 °C for 3 h. The reaction was quenched with water (100 mL) and the product precipitated as a yellow solid. This solid was separated by filtration and washed with distilled water. The product was purified using standard column chromatography (SiO_2) with cyclohexane and ethyl acetate (v:v = 4:1) as solvents. The product was dried *in vacuo* and obtained as a yellow solid. (Yield 172 mg, 94%). Molecular mass: 528.64 g/mol, Molecular formula: $C_{29}H_{24}N_2O_4S_2$, ^{1}H-NMR (300 MHz, DMSO-d_6) δ = 7.57 – 7.50 (*m*, 4H), 7.44 (*s*, 1H), 7.38 (*s*, 1H), 7.12 (*d*, *J* = 8.9 Hz, 2H), 7.05 (*d*, *J* = 8.9 Hz, 2H), 4.88 (*d*, *J* = 2.4 Hz, 2H), 4.75 (*s*, 2H), 3.62 (*t*, *J* = 2.4 Hz, 1H), 1.42

(*s*, 9H) ppm. ^{13}C-NMR (151 MHz, DMSO-*d*$_6$) δ = 167.50, 159.21, 158.65, 140.98, 140.71, 136.03, 135.95, 133.39, 133.12, 120.30, 120.00, 116.76, 116.53, 115.48, 115.39, 115.24, 81.62, 78.78, 78.70, 65.08, 55.70, 27.69 ppm. (2 C's of the central aromatic unit overlap) IR (ATR, $\tilde{\nu}$/cm^{-1}): 3302, 2982, 2934, 2226, 2126, 1721, 1587, 1570, 1489, 1449, 1408, 1395, 1370, 1341, 1312, 1302, 1290, 1261, 1223, 1175, 1155, 1109, 1101, 1090, 1063, 1013, 941, 930, 889, 831, 814, 781, 723, 685, 644, 606. HR-ESI-pos (MeOH): m/z = 551.1067. (calcd. 551.1070 for [$C_{29}H_{24}N_2O_4S_2$ + Na]$^+$).

2-(4-((2,5-dicyano-4-((4-(prop-2-yn-1-yloxy)phenyl)thio)phenyl)thio)phenoxy)acetic acid (G)

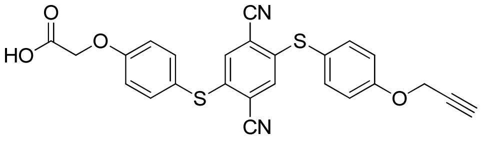

Compound **(F)** (151 mg, 0.29 mmol) was added to a 50 mL round bottom flask followed by the addition of DCM (10 mL). Than trifluoroacetic acid (5 mL, 65.34 mmol) was added to the reaction. The solution was stirred at RT for 3 h. The solvents were removed and the product was dried *in vacuo*. The product obtained as a yellow solid. (Yield 137 mg, quant.). Molecular mass: 472.53 g/mol, Molecular formula: $C_{25}H_{16}N_2O_4S_2$, ^{1}H-NMR (300 MHz, DMSO-*d*$_6$) δ = 13.07 (*s*, 1H), 7.56 – 7.50 (*m*, 4H), 7.44 (*s*, 1H), 7.41 (*s*, 1H), 7.12 (*d*, *J* = 8.8 Hz, 2H), 7.06 (*d*, *J* = 8.8 Hz, 2H), 4.88 (*d*, *J* = 2.4 Hz, 2H), 4.76 (s, 2H), 3.62 (*t*, *J* = 2.3 Hz, 1H) ppm. ^{13}C-NMR (151 MHz, DMSO-*d*$_6$) δ = 169.82, 159.26, 158.65, 140.92, 140.74, 136.03, 135.96, 133.35, 133.24, 120.30, 119.90, 116.76, 116.48, 115.48, 115.43, 115.27, 78.78, 78.70, 64.57, 55.70 ppm. (2 C's of the central aromatic unit overlap) IR (ATR, $\tilde{\nu}$/cm^{-1}): 3285, 3080, 2232, 2131, 1765, 1589, 1572, 1491, 1449, 1435, 1410, 1387, 1344, 1302, 1287, 1244, 1157, 1103, 1090, 1072, 1032, 1011, 959, 901, 880, 829, 770, 723, 708, 692, 646, 619. HR-ESI-pos (MeOH): m/z = 495.0441. (calcd. 495.0444 for [$C_{25}H_{16}N_2O_4S_2$ + Na]$^+$).

***tert*-butyl (2-((tert-butoxycarbonyl)amino)ethyl)(2-(2-(4-((2,5-dicyano-4-((4-(prop-2-yn-1-yloxy)phenyl)thio)phenyl)thio)phenoxy)acetamido)ethyl)carbamate (H)**

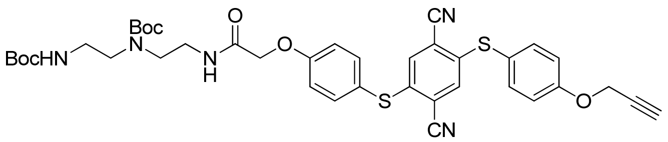

Compound **(G)** (70 mg, 0.15 mmol) was added to a 25 mL round bottom flask followed by the addition of DMF (5 mL). A solution of 1-ethyl-3-(3-dimethylaminopropyl)carbodiimide (EDCI) (31 mg, 0.16 mmol), 1-Hydroxybenzotriazole (HOBt) (25 mg, 0.16 mmol) and *N*-Methylmorpholine (NMM) (33 µL, 0.30 mmol) in DMF (3 mL) were added to compound **(G)**. After 15 minutes stirring at RT the 1,4-bis-boc-1,4,7-triazaheptane (49 mg, 0.13 mmol) in DMF (2 mL) was added to the reaction mixture. The solution was stirred at RT for 48 h. The reaction was quenched with water (50 mL) and extracted several times with ethylacetate. The combined organic layer was dried over $MgSO_4$ and the solvent was evaporated in vacuo. The product was purified using standard column chromatography with dichloromethane and methanol (v:v = 98:2) as solvents. The product was dried *in vacuo* and obtained as a yellow solid. (Yield 68 mg, 61%). Molecular mass: 757.92 g/mol, Molecular formula: $C_{39}H_{43}N_5O_7S_2$, ^{1}H-NMR (300 MHz, DMSO-*d*$_6$) δ = 8.18 (*s*, 1H), 7.54 (*dd*, *J* = 8.7, 1.8 Hz, 4H), 7.44 (*s*, 1H), 7.40 (*s*, 1H), 7.11 (*t*, *J* = 9.0 Hz, 4H), 6.78 (*s*, 1H), 4.88 (*d*, *J* = 2.3 Hz, 2H), 4.53 (*s*, 2H), 3.61 (*t*, *J* = 2.3 Hz, 1H), 3.24 (*s*, 4H), 3.16 (*t*, *J* = 6.3 Hz, 2H), 3.02 (*d*, *J* = 4.6 Hz, 2H), 1.38 (*s*, 9H), 1.36 (*s*, 9H) ppm. ^{13}C-NMR (151 MHz, DMSO-*d*$_6$) δ = 167.29, 159.16, 158.65, 155.57, 154.98, 140.92, 140.73, 136.02, 135.95, 133.37, 133.18, 120.28, 120.11, 120.06, 116.75, 116.70, 115.49, 115.41, 115.24, 78.77, 78.69, 77.55, 67.01, 66.95, 55.70, 46.94, 46.02, 38.50, 37.18, 28.22, 28.01 ppm. IR (ATR, $\tilde{\nu}$/cm^{-1}): 3285, 3082, 2976, 2930, 2224, 2120, 1674, 1589, 1572, 1491, 1449, 1412, 1391, 1366, 1290, 1231, 1173, 1152, 1103, 1018, 1009, 926, 891, 831, 808, 773, 723, 692, 646. HR-ESI-pos (MeOH): m/z = 780.2496. (calcd. 780.2496 for [$C_{39}H_{43}N_5O_7S_2$ + Na]$^+$).

***N*^{1}-(2-(2-(4-((2,5-dicyano-4-((4-(prop-2-yn-1-yloxy)phenyl)thio)phenyl)thio)phenoxy)acetamido)ethyl)ethane-1,2-diaminium chloride (2)**

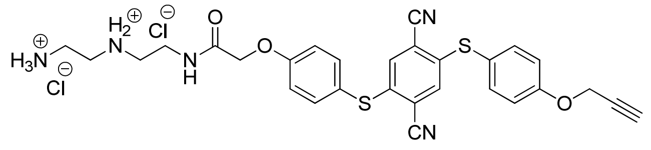

Compound **(H)** (20 mg, 0.026 mmol) was added to a 25 mL round bottom flask followed by the addition of MeOH (5 mL). The solution was cooled to 0 °C. Than acetyl chloride (0.2 mL, 2.81 mmol) was added via a syringe. The solution was stirred at 0 °C for 2 h and at RT for 20 h. The solvents were removed *in vacuo*. The solid was suspended in water and dried by freeze-drying. The product obtained as a yellow solid. (Yield 16 mg, 96%). Molecular mass: 630.61 g/mol, Molecular formula: $C_{29}H_{29}Cl_2N_5O_3S_2$, ^{1}H-NMR (600 MHz, DMSO-d_6) δ = 9.47 (*s*, 2H), 8.48 (*d*, *J* = 4.9 Hz, 1H), 8.31 (*s*, 3H), 7.54 (*d*, *J* = 8.6 Hz, 4H), 7.44 (*s*, 1H), 7.40 (*s*, 1H), 7.15 (*d*, *J* = 8.8 Hz, 2H), 7.13 (*d*, *J* = 8.7 Hz, 2H), 4.88 (*d*, *J* = 2.3 Hz, 2H), 4.62 (*s*, 2H), 3.63 (*t*, *J* = 2.3 Hz, 1H), 3.49 (*dd*, *J* = 11.9, 6.0 Hz, 2H), 3.22 (*d*, *J* = 5.2 Hz, 2H), 3.19 (*d*, *J* = 4.5 Hz, 2H), 3.09 (*t*, *J* = 6.1 Hz, 2H) ppm. ^{13}C-NMR (151 MHz, DMSO-d_6) δ = 168.10, 159.11, 158.65, 140.90, 140.74, 136.02, 135.96, 133.40, 133.22, 120.28, 120.15, 116.77, 115.48, 115.42, 115.29, 115.25, 78.78, 78.72, 66.94, 55.71, 46.28, 44.07, 35.28, 34.85 ppm. (2 C's of the central aromatic unit overlap) IR (ATR, $\tilde{\nu}$/cm^{-1}): 3283, 2918, 2851, 2780, 2685, 2380, 2224, 2118, 2087, 1894, 1871, 1736, 1618, 1665, 1587, 1572, 1541, 1535, 1489, 1449, 1408, 1371, 1344, 1292, 1260, 1219, 1173, 1103, 1047, 1017, 1009, 936, 926, 893, 831, 806, 777, 723, 706, 692, 646, 625. HR-ESI-pos (MeOH): m/z = 558.1627. (calcd. 558.1628 for $[C_{29}H_{27}N_5O_3S_2 + H]^+$).

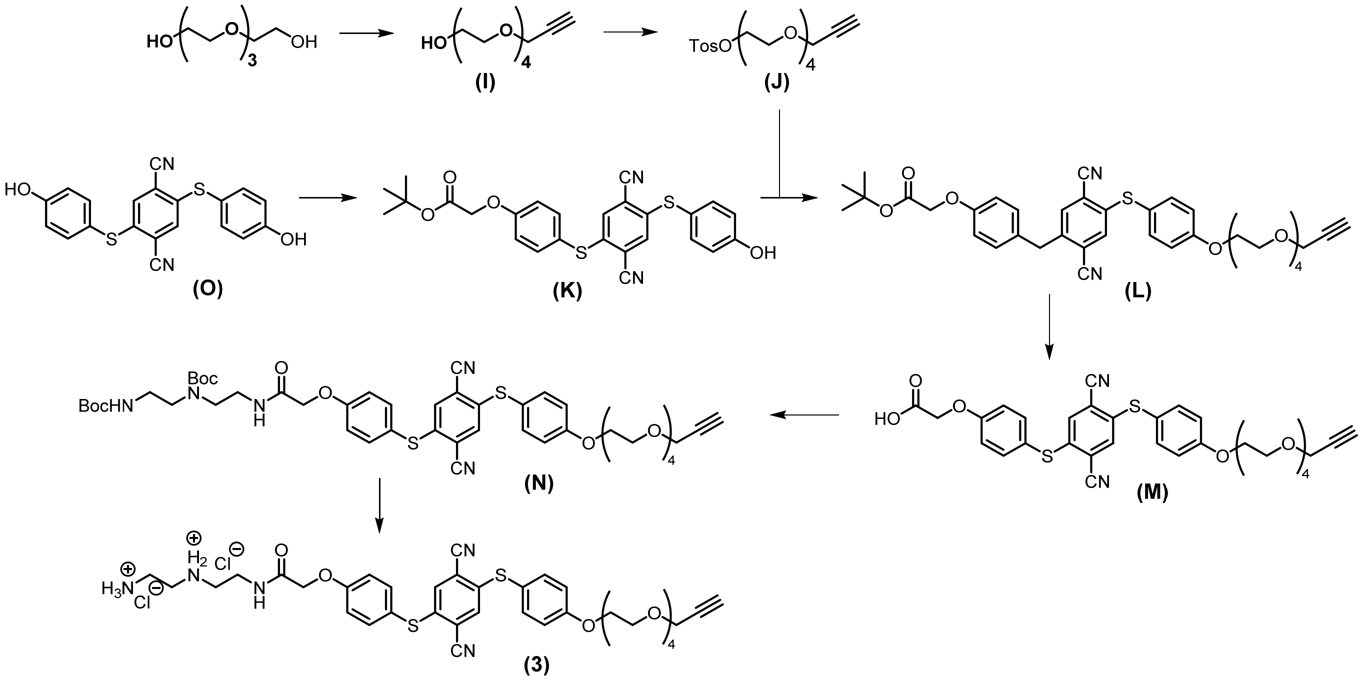

Scheme S3: Synthetic routes to the clickable compound (**3**).

3,6,9,12-tetraoxapentadec-14-yn-1-ol (I)[4]

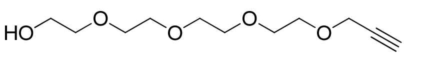

Compound (**I**) was synthesized according to a slightly modified literature procedure.[4] Tetraethylene glycol (3.87 g, 19.90 mmol) was added under argon to a 100 mL round bottom flask followed by the addition of dry THF (40 mL). The solution was cooled to 0 °C. Then sodium hydride (60% dispersion in mineral oil, 1.06 g, 26.50 mmol) was added in portions carefully. After no more gas evolved, propargyl bromide (3.31 g, 22.27 mmol) was added dropwise at 0 °C via syringe injection. The solution was stirred at RT for 20 h. The reaction mixture was filtered over celite and the solvents of the clear solution were evaporated in vacuo. The product was purified using standard column chromatography with dichloromethane and methanol (v:v = 99:1 → v:v = 95:5) as solvents. The product was dried *in vacuo* and obtained as a yellowish oil. (Yield 7.78 g, 39%). Molar mass: 232.27 g/mol, Molecular formula: $C_{11}H_{20}O_5$, ^{1}H-NMR (300 MHz, $CDCl_3$) δ = 4.20 (*d*, *J* = 2.3, 2H,), 3.73-3.63 (*m*, 14H), 3.62-3.59 (*m*, 2H), 2.46 (*t*, *J* = 5.3 Hz, 1H), 2.42 (*t*, *J* = 2.4 Hz, 1H) ppm. ^{13}C-NMR (75 MHz, $CDCl_3$) δ = 79.8, 74.6, 72.5, 70.8, 70.8, 70.7, 70.6, 70.6, 69.3, 61.9, 58.6 ppm. IR (ATR, $\tilde{\nu}$/cm^{-1}): 3418, 3260, 2868, 2112, 1717, 1593, 1493, 1449, 1348, 1287, 1246, 1177, 1090, 1032, 934, 920, 883, 835, 669. HR-ESI-pos (MeOH): m/z = 255.1203. (calcd. 255.1203 for $[C_{11}H_{20}O_5 + Na]^+$).

3,6,9,12-tetraoxapentadec-14-yn-1-yl 4-methylbenzenesulfonate (J)[4]

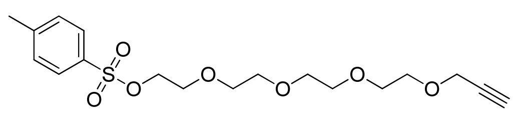

Compound (**J**) was synthesized according to a slightly modified literature procedure.[4] Compound **(I)** (1.81 g, 7.78 mmol) was added to a 100 mL round bottom flask followed by the addition of THF (10 mL) and an aqueous NaOH solution (1.10 g in 11 mL). The solution was cooled to 0 °C. Than a solution of *p*-toluenesulfonylchloride (3.00 g, 15.72 mmol) in THF (15 mL) was added. The reaction was stirred at RT for 48 h. After the addition of DCM (80 mL) the mixture was washed with water. The organic layer was dried over $MgSO_4$ and the solvent was evaporated *in vacuo*. The product was purified using standard column chromatography with cyclohexane and ethyl acetate (v:v = 1:1 → v:v = 1:2) as solvents. The product was dried *in vacuo* and obtained as a yellowish oil. (Yield 1.84 g, 61%). Molecular mass: 386.46 g/mol, Molecular formula: $C_{18}H_{26}O_7S$, ^{1}H-NMR (300 MHz, $CDCl_3$) δ = 7.80 (*d*, *J* = 8.0 Hz, 2H), 7.34 (*d*, *J* = 8.1 Hz, 2H), 4.20 (*d*, J = 2.4 Hz, 2H), 3.69-3.56 (*m*, 16H), 2.45 (*s*, 3H), 2.42 (*t*, *J* = 2.4 Hz, 1H) ppm. ^{13}C-NMR (75 MHz, $CDCl_3$) δ = 145.3, 133.7, 130.4, 128.5, 80.2, 75.1, 71.3, 71.2, 71.1, 71.0, 69.8, 69.7, 69.3, 59.0, 22.2 ppm. IR (ATR, $\tilde{\nu}$/cm^{-1}): 3277, 2868, 2114, 1719, 1597, 1495, 1451, 1398, 1350, 1290, 1246, 1188, 1175, 1094, 1011, 916, 816, 773, 704, 662. HR-ESI-pos (MeOH): m/z = 409.1292. (calcd. 409.1291 for $[C_{18}H_{26}O_7S + Na]^+$).

***tert*-butyl 2-(4-((2,5-dicyano-4-((4-hydroxyphenyl)thio)phenyl)thio)phenoxy)acetate (K)**

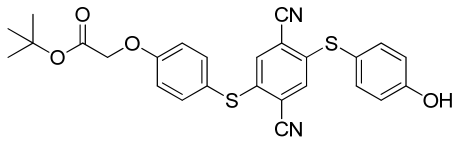

Compound **(O)** (0.50 g, 3.49 mmol) and potassium carbonate (1.13 g, 8.21 mmol) were added under argon to a 100 mL round bottom flask followed by the addition of dry DMF (20 mL). The solution was stirred at 45 °C for 10 minutes. Then *tert*-butyl bromoacetate (0.18 mL, 1.26 mmol) in DMF (30 mL) was added dropwise. The suspension was stirred at 45 °C for 18 h. The solvent of the reaction was evaporated in vacuum. The solid was treated with 2 M HCl to protonate the product. The precipitate was separated by filtration and washed with distilled water. The product was purified using standard column chromatography (SiO_2) with cyclohexane and acetone (v:v = 3:1) as solvents. The product was dried *in vacuo* and obtained as a yellow solid. (Yield 260 mg, 42%). Molecular mass: 490.59 g/mol, Molecular formula: $C_{26}H_{22}N_2O_4S_2$, ^{1}H-NMR (300 MHz, DMSO-*d*$_6$) δ = 10.15 (*s*, 1H), 7.51 (*d*, *J* = 8.8 Hz, 2H), 7.42 (*d*, *J* = 7.8 Hz, 3H), 7.26 (*s*, 1H), 7.04 (*d*, *J* = 8.9 Hz, 2H), 6.91 (*d*, *J* = 8.7 Hz, 2H), 4.74 (*s*, 2H), 1.42 (*s*, 9H) ppm. ^{13}C-NMR (75 MHz, DMSO-d$_6$) δ = 167.43, 159.52, 159.08, 142.29, 139.88, 136.73, 135.77, 133.40, 132.15, 120.36, 117.39, 116.45, 116.34, 115.67, 115.25, 115.11, 114.36, 81.55, 65.08, 27.65 ppm. IR (ATR, $\tilde{\nu}$/cm^{-1}): 3385, 3080, 2924, 2238, 2224, 1898, 1748, 1724, 1586, 1493, 1447, 1410, 1393, 1366, 1344, 1325, 1308, 1290, 1281, 1263, 1221, 1150, 1105, 1096, 1076, 1065, 1040, 1009, 947, 895, 880, 831, 816, 802, 781, 748, 733, 723, 708, 646. HR-ESI-pos (MeOH): m/z = 513.0916. (calcd. 513.0913 for $[C_{26}H_{22}N_2O_4S_2 + Na]^+$).

***tert*-butyl 2-(4-((4-((4-(3,6,9,12-tetraoxapentadec-14-yn-1-yloxy)phenyl)thio)-2,5-dicyanophenyl)thio)phenoxy)acetate (L)**

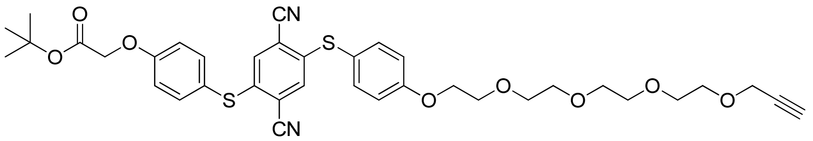

Compound **(K)** (178 mg, 0.36 mmol), compound **(J)** (140 mg, 0.36 mmol) and potassium carbonate (192 mg, 1.39 mmol) were added to a 50 mL round bottom flask followed by the addition of dry DMF (10 mL). The suspension was stirred at 50 °C for 18h. The reaction was quenched with water (100 mL). After the precipitation of the product the solid was separated by filtration and washed with distilled water. The product was purified using standard column chromatography with cyclohexane and ethyl acetate (v:v = 2:1 → v:v = 1:1) as solvents. The product was dried *in vacuo* and obtained as a yellow solid. (Yield 162 mg, 64%). Molecular mass: 704.85 g/mol, Molecular formula: $C_{37}H_{40}N_2O_8S_2$, ^{1}H-NMR (300 MHz, DMSO-*d*$_6$) δ = 7.52 (*dd*, *J* = 8.7, 1.5 Hz, 4H), 7.38 (*s*, 2H), 7.09 (*d*, *J* = 8.8 Hz, 2H), 7.05 (*d*, *J* = 8.8 Hz, 2H), 4.74 (*s*, 2H), 4.18 – 4.14 (*m*, 2H), 4.13 (*d*, *J* = 2.4 Hz, 2H), 3.79 – 3.73 (*m*, 2H), 3.62 – 3.57 (*m*, 2H), 3.56 – 3.50 (*m*, 10H), 3.41 (*t*, *J* = 2.4 Hz, 1H), 1.42 (*s*, 9H) ppm. ^{13}C-NMR (75 MHz, DMSO-d$_6$) δ = 167.44, 160.03, 159.15, 141.19, 140.62, 136.18, 135.92, 133.18, 132.97, 120.10, 119.14, 116.49, 116.45, 115.46, 115.19, 115.17, 115.12, 81.57, 80.29, 77.01, 69.89, 69.76, 69.46, 68.74, 68.48, 67.51, 67.45, 65.08, 57.45, 27.66 ppm (2 CH_2 groups of the tetraethyleneglycol chains overlap). IR (ATR, $\tilde{\nu}$/cm^{-1}): 3285, 3080, 2972, 2870, 2224, 2112, 1748, 1726, 1589, 1493, 1472, 1447, 1410, 1393, 1368, 1344, 1325, 1306, 1290, 1254, 1221, 1177, 1140, 1103, 1074, 1055, 1009, 947, 918, 885, 868, 837, 829, 802, 781, 746, 731, 721, 706, 675, 652, 646. HR-ESI-pos (MeOH): m/z = 727.2120. (calcd. 727.2118 for $[C_{26}H_{22}N_2O_4S_2 + Na]^+$).

2-(4-((4-((4-(3,6,9,12-tetraoxapentadec-14-yn-1-yloxy)phenyl)thio)-2,5-dicyanophenyl)thio)phenoxy)acetic acid (M)

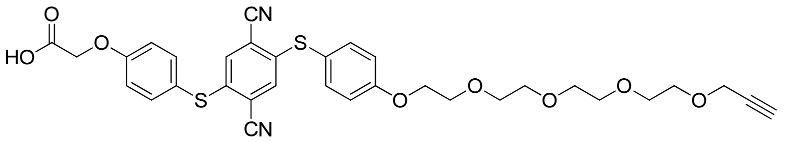

Compound **(L)** (141 mg, 0.20 mmol) was added to a 50 mL round bottom flask followed by the addition of DCM (10 mL). Then trifluoroacetic acid (5 mL, 65.34 mmol) was added to the reaction. The solution was stirred at RT for 3 h. The solvents were removed and the product was dried *in vacuo*. The product obtained as a yellow solid. (Yield 132 mg, quant.). Molecular mass: 648.75 g/mol, Molecular formula: $C_{33}H_{32}N_2O_8S_2$, ^{1}H-NMR (300 MHz, DMSO-d_6) δ = 13.08 (*s*, 1H), 7.52 (*d*, *J* = 8.8 Hz, 4H), 7.42 (*s*, 1H), 7.38 (*s*, 1H), 7.09 (*d*, *J* = 8.8 Hz, 2H), 7.06 (*d*, *J* = 8.8 Hz, 2H), 4.76 (*s*, 2H), 4.18 – 4.14 (*m*, 2H), 4.13 (*d*, *J* = 2.4 Hz, 2H), 3.79 – 3.74 (*m*, 2H), 3.62 – 3.58 (*m*, 2H), 3.57 – 3.50 (*m*, 10H), 3.40 (*t*, *J* = 2.4 Hz, 1H). ^{13}C-NMR (151 MHz, DMSO-d_6) δ = 169.82, 160.07, 159.23, 141.28, 140.60, 136.25, 135.98, 133.32, 132.94, 120.00, 119.13, 116.47, 115.51, 115.27, 115.25, 115.12, 80.34, 77.10, 69.92, 69.80, 69.79, 69.76, 69.49, 68.78, 68.51, 67.47, 64.58, 57.49 ppm. (2 CH_2 groups of the tetraethyleneglycol chains overlap) IR (ATR, $\tilde{\nu}$/cm^{-1}): 3239, 3082, 2918, 2870, 2239, 2114, 1765, 1591, 1574, 1493, 1447, 1435, 1410, 1344, 1289, 1248, 1159, 1103, 1072, 1011, 916, 899, 878, 827, 777, 723, 694, 646, 621 ppm. HR-ESI-pos (MeOH): m/z = 671.1492. (calcd. 671.1492 for $[C_{33}H_{32}N_2O_8S_2 + Na]^+$).

***tert*-butyl (2-(2-(4-((4-((4-(3,6,9,12-tetraoxapentadec-14-yn-1-yloxy)phenyl)thio)-2,5-dicyanophenyl)thio)phenoxy)acetamido)ethyl)(2-((tert-butoxycarbonyl)amino)ethyl)carbamate (N)**

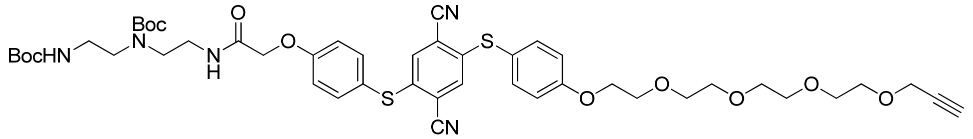

Compound **(M)** (80 mg, 0.12 mmol) was added to a 25 mL round bottom flask followed by the addition of DMF (5 mL). A solution of 1-ethyl-3-(3-dimethylaminopropyl)carbodiimide (EDCI) (26 mg, 0.14 mmol), 1-Hydroxybenzotriazole (HOBt) (20 mg, 0.14 mmol) and *N*-Methylmorpholine (NMM) (27 µL, 0.25 mmol) in DMF (3 mL) were added to compound **(M)**. After 15 minutes stirring at RT the 1,4-bis-boc-1,4,7-triazaheptane (41 mg, 0.14 mmol) in DMF (2 mL) was added to the reaction mixture. The solution was stirred at RT for 48 h. The reaction was quenched with water (50 mL) and extracted several times with ethyl acetate. The combined organic layer was dried over $MgSO_4$ and the solvent was evaporated in vacuo. The product was purified using standard column chromatography (SiO_2) with dichloromethane and methanol (v:v = 98:2) as solvents. The product was dried *in vacuo* and obtained as a yellow solid. (Yield 99 mg, 86%). Molecular mass: 934.13 g/mol, Molecular formula: $C_{47}H_{59}N_5O_{11}S_2$, ^{1}H-NMR (300 MHz, DMSO-d_6) δ = 8.18 (*s*, 1H), 7.52 (*dd*, *J* = 8.7, 4.3 Hz, 4H), 7.41 (*s*, *J* = 10.4 Hz, 1H), 7.38 (*s*, 1H), 7.09 (*d*, *J* = 8.7 Hz, 4H), 6.81 (*s*, 1H), 4.53 (*s*, 2H), 4.18 – 4.14 (*m*, 2H), 4.13 (*d*, *J* = 2.5 Hz, 2H), 3.79 – 3.74 (*m*, 2H), 3.62 – 3.57 (*m*, 2H), 3.56 – 3.50 (*m*, 10H), 3.40 (*t*, *J* = 2.3 Hz, 1H), 3.24 (*s*, 4H), 3.16 (*t*, *J* = 6.4 Hz, 2H), 3.03 (*s*, 2H), 1.38 (*s*, 9H), 1.36 (*s*, 9H) ppm. ^{13}C-NMR (151 MHz, DMSO-d_6) δ = 167.28, 160.06, 159.13, 155.57, 154.97, 154.68, 141.26, 140.59, 136.24, 135.97, 133.25, 132.95, 120.20, 120.16, 119.11, 116.68, 116.47, 115.48, 115.24, 115.22, 115.11, 80.32, 78.66, 78.61, 77.54, 77.09, 69.91, 69.79, 69.78, 69.75, 69.48, 68.77, 68.50, 67.47, 67.01, 66.94, 57.48, 28.22, 28.00 ppm. IR (ATR, $\tilde{\nu}$/cm^{-1}): 3283, 2924, 2226, 2120, 1676, 1591, 1572, 1530, 1491, 1449, 1410, 1391, 1366, 1287, 1244, 1173, 1146, 1101, 1053, 1007, 953, 883, 829, 775, 708, 646. HR-ESI-pos (MeOH): m/z = 956.3548. (calcd. 956.3545 for $[C_{47}H_{59}N_5O_{11}S_2 + Na]^+$).

***N*^{1}-(2-(2-(4-((4-((4-(3,6,9,12-tetraoxapentadec-14-yn-1-yloxy)phenyl)thio)-2,5-dicyanophenyl)thio)phenoxy)acetamido)ethyl)ethane-1,2-diaminium chloride (3)**

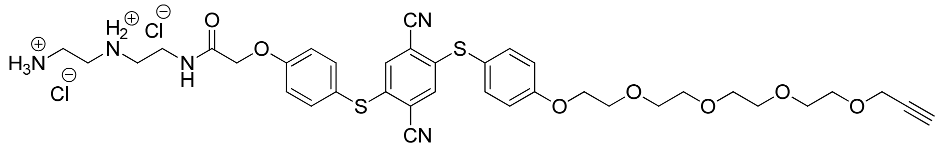

Compound **(N)** (10 mg, 0.011 mmol) was added to a 25 mL round bottom flask followed by the addition of MeOH (5 mL). The solution was cooled to 0 °C. Then acetyl chloride (0.1 mL, 1.41 mmol) was added via syringe. The solution was stirred at 0 °C for 2 h and at RT for 20 h. The solvents were removed in vacuum. The solid was suspended in water and dried by freeze-drying. The product was obtained as a yellow solid. (Yield 9 mg, quant.). Molecular mass: 806.82 g/mol, Molecular formula:

$C_{37}H_{45}Cl_2N_5O_7S_2$, ^{1}H-NMR (600 MHz, DMSO-d_6) δ = 9.43 (*s*, 2H), 8.47 (*t*, *J* = 5.8 Hz, 1H), 8.27 (*s*, 3H), 7.54 (*d*, *J* = 8.8 Hz, 2H), 7.51 (*d*, *J* = 8.8 Hz, 2H), 7.41 (*s*, 1H), 7.39 (*s*, 1H), 7.15 (*d*, *J* = 8.8 Hz, 2H), 7.10 (*d*, *J* = 8.8 Hz, 2H), 4.61 (*s*, 2H), 4.17 – 4.14 (*m*, 2H), 4.13 (*d*, *J* = 2.4 Hz, 2H), 3.77 – 3.75 (*m*, 2H), 3.59 (*dd*, *J* = 5.9, 3.5 Hz, 2H), 3.55 – 3.47 (*m*, 12H), 3.41 (*t*, *J* = 2.4 Hz, 1H), 3.22 (*d*, *J* = 5.1 Hz, 2H), 3.18 (*d*, *J* = 4.8 Hz, 2H), 3.10 (*t*, *J* = 5.2 Hz, 2H) ppm. ^{13}C-NMR (151 MHz, DMSO-d_6) δ = 168.13, 160.07, 159.06, 141.28, 140.56, 136.24, 135.97, 133.28, 132.96, 120.26, 119.09, 116.75, 116.48, 115.49, 115.26, 115.25, 115.10, 80.33, 77.11, 69.91, 69.79, 69.78, 69.75, 69.48, 68.77, 68.50, 67.47, 66.94, 57.48, 46.31, 44.07, 35.28, 34.84 ppm. IR (ATR, $\tilde{\nu}$/cm^{-1}): 3277, 2870, 2700, 2575, 2440, 2384, 2224, 2112, 2087, 1900, 1748, 1672, 1611, 1589, 1572, 1528, 1493, 1449, 1410, 1375, 1346, 1290, 1248, 1229, 1177, 1101, 1094, 1053, 1007, 939, 883, 831, 721, 708, 675, 652, 646, 631, 608. HR-ESI-pos (MeOH): m/z = 378.6281. (calcd. 378.6284 for $[C_{37}H_{43}N_5O_7S_2 + Na + H]^{2+}$).

3. NMR Spectra of the final compounds

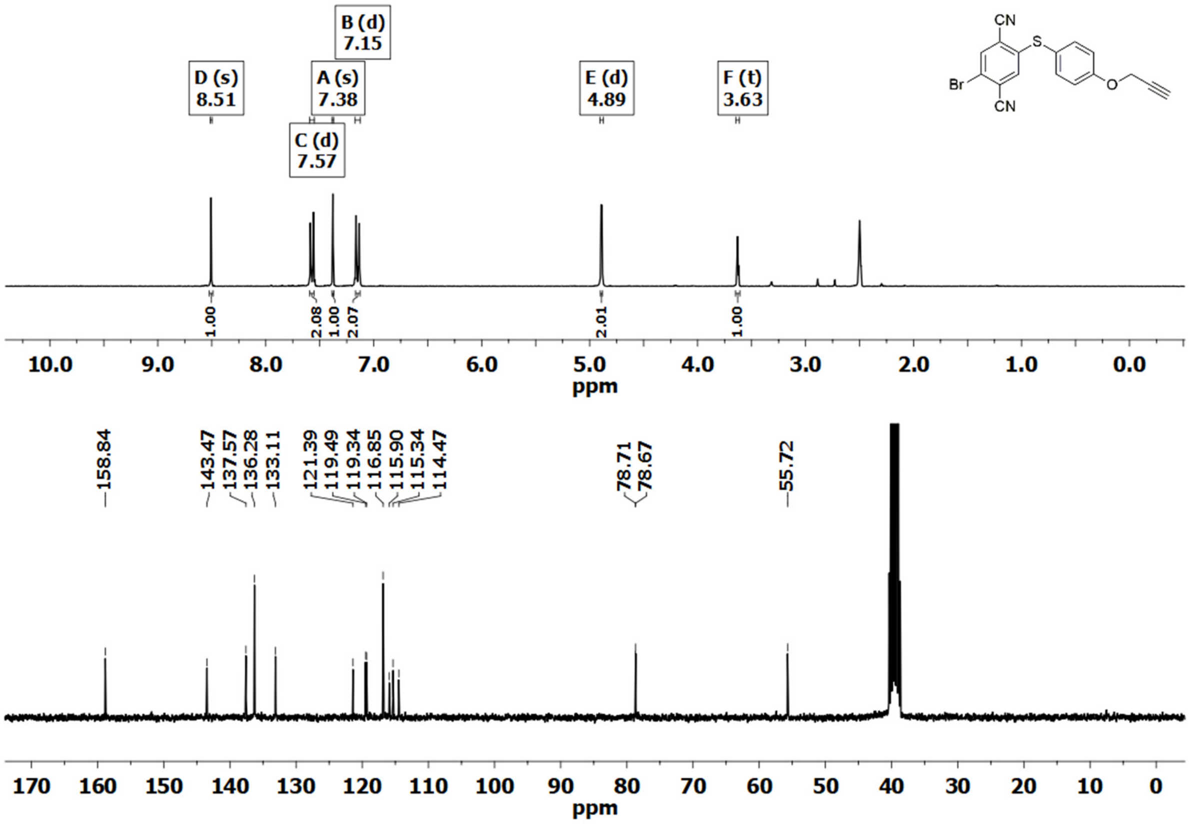

Figure S1: ^{1}H- and ^{13}C-NMR of compound **(C)**. * = water. (DMSO-d_6).

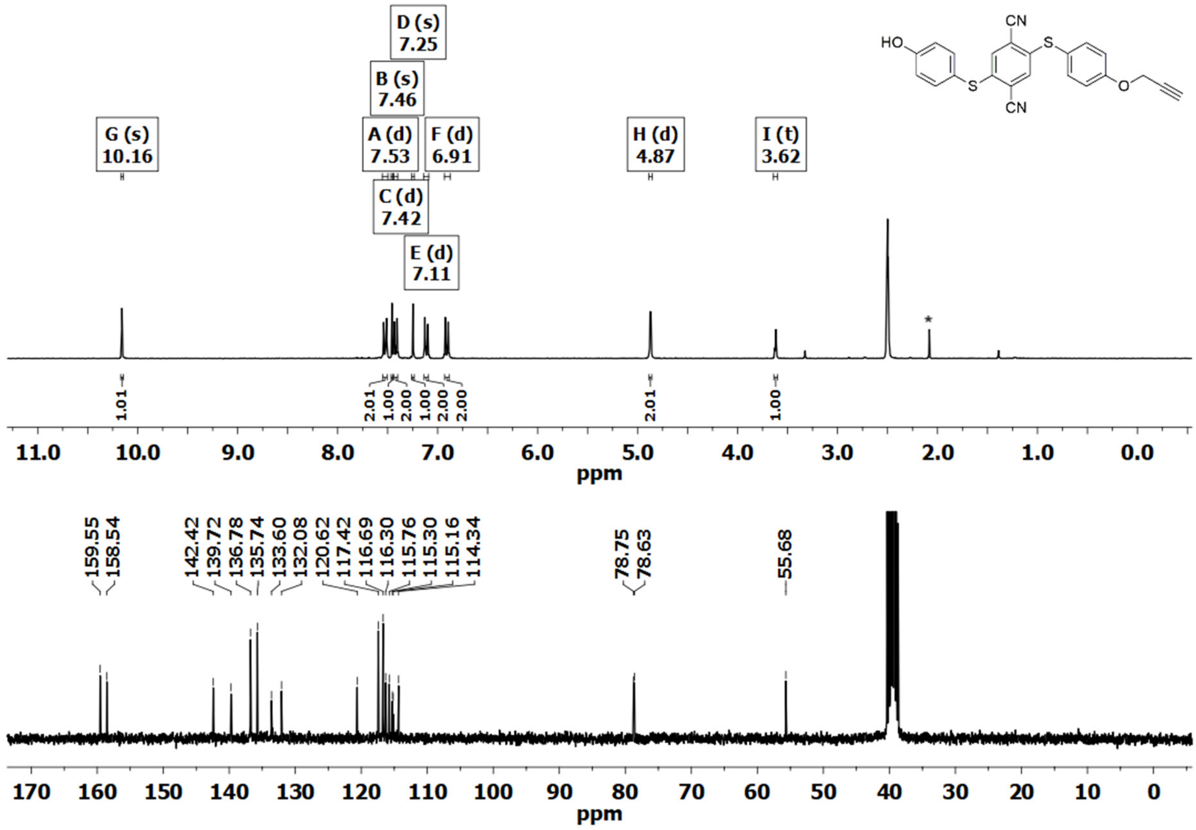

Figure S2: ^{1}H and ^{13}C NMR of compound **(D)**. * = acetone. (DMSO-d_6).

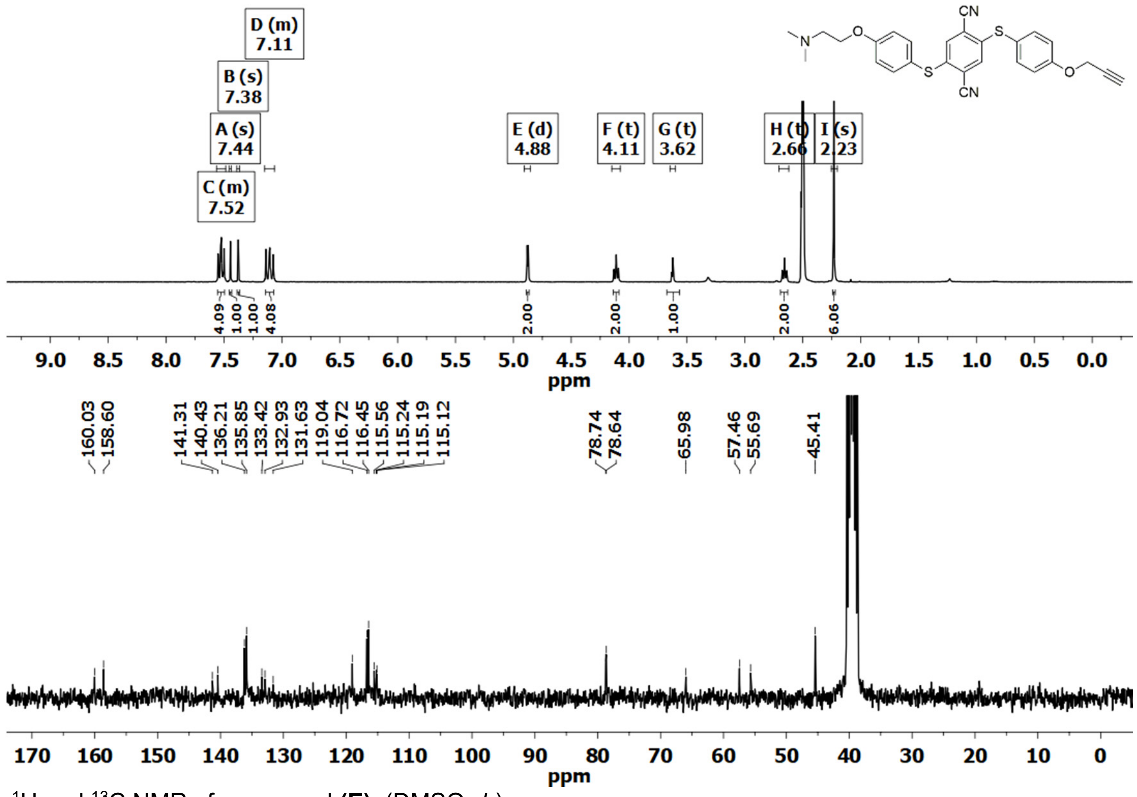

Figure S3: ^{1}H and ^{13}C NMR of compound **(E)**. (DMSO-d_6).

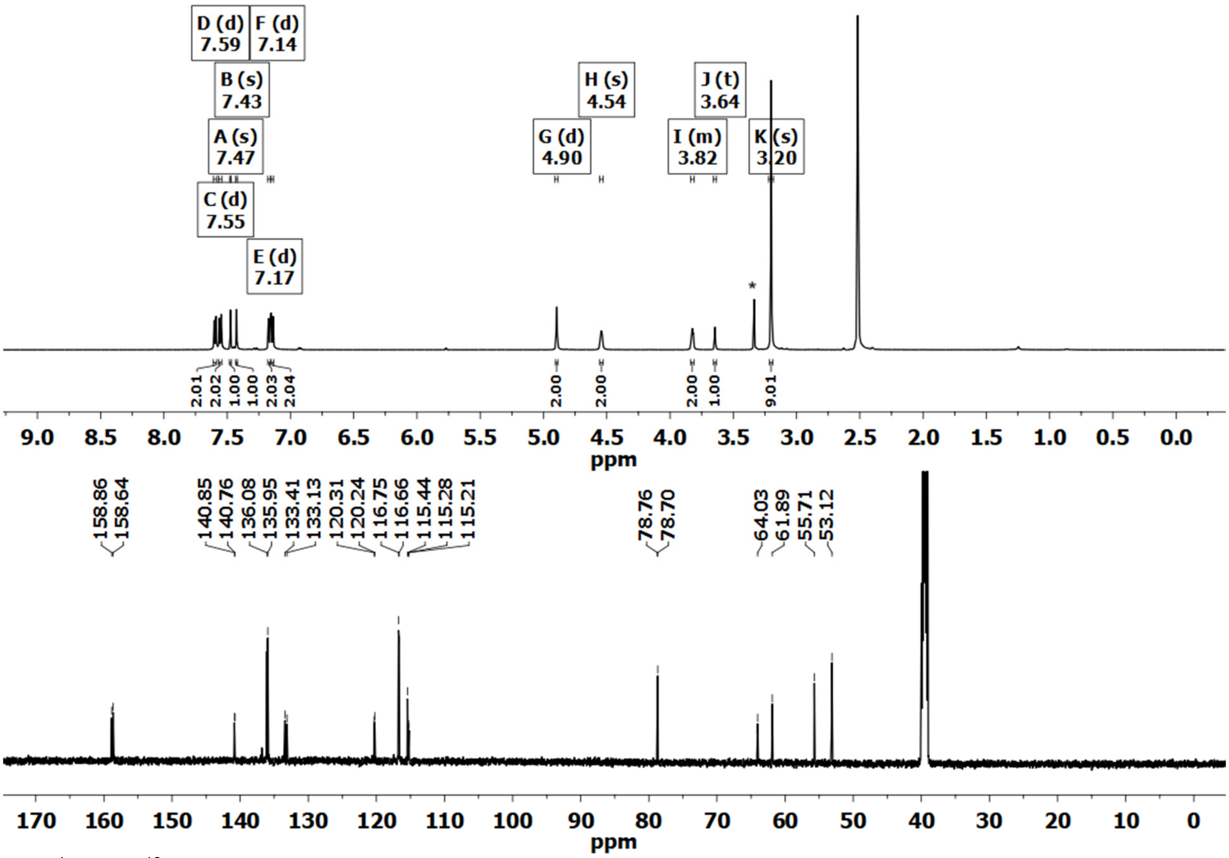

Figure S4: ^{1}H- and ^{13}C-NMR of compound **(1)**. * = water. (DMSO-d_6)

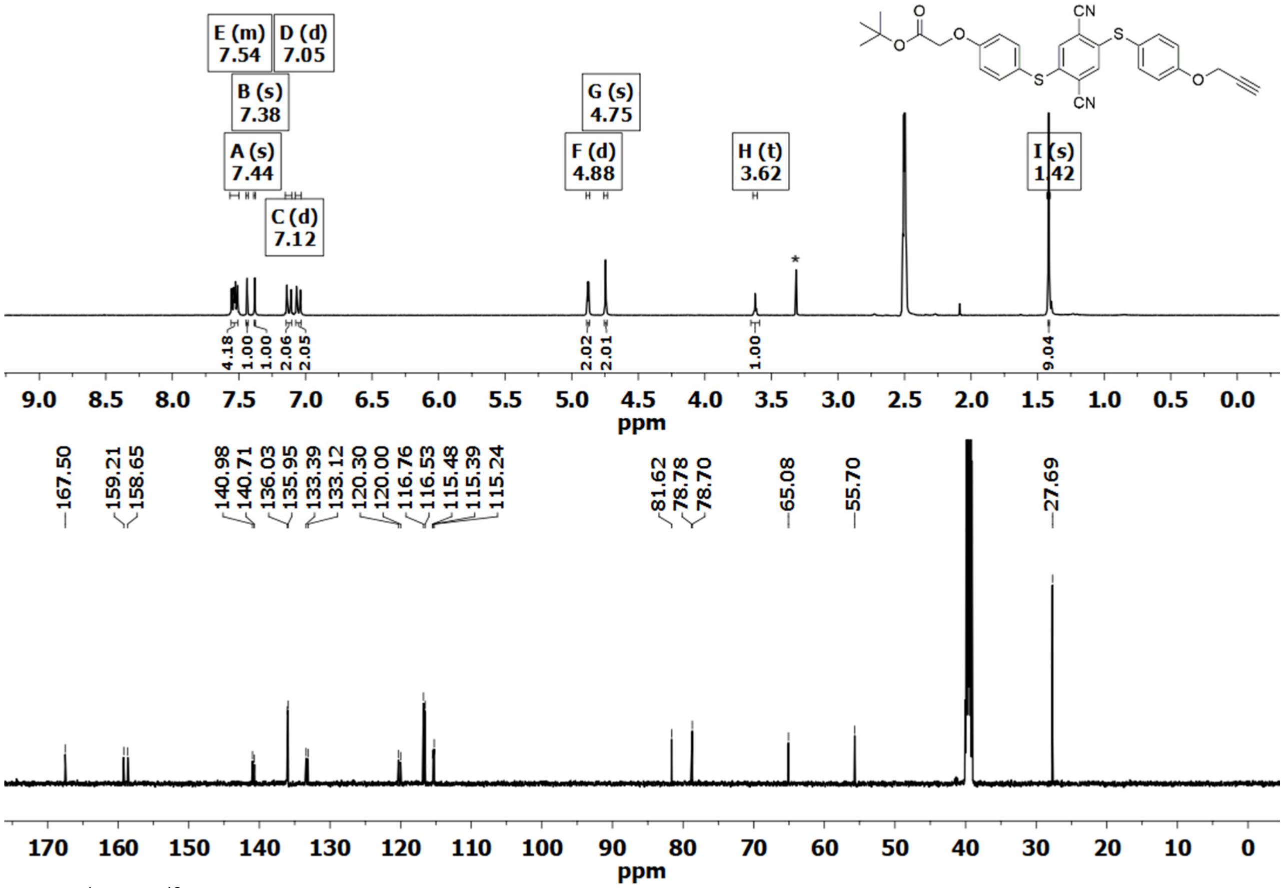

Figure S5: ^{1}H and ^{13}C NMR of compound **(F)**. * = water. (DMSO-*d*6)

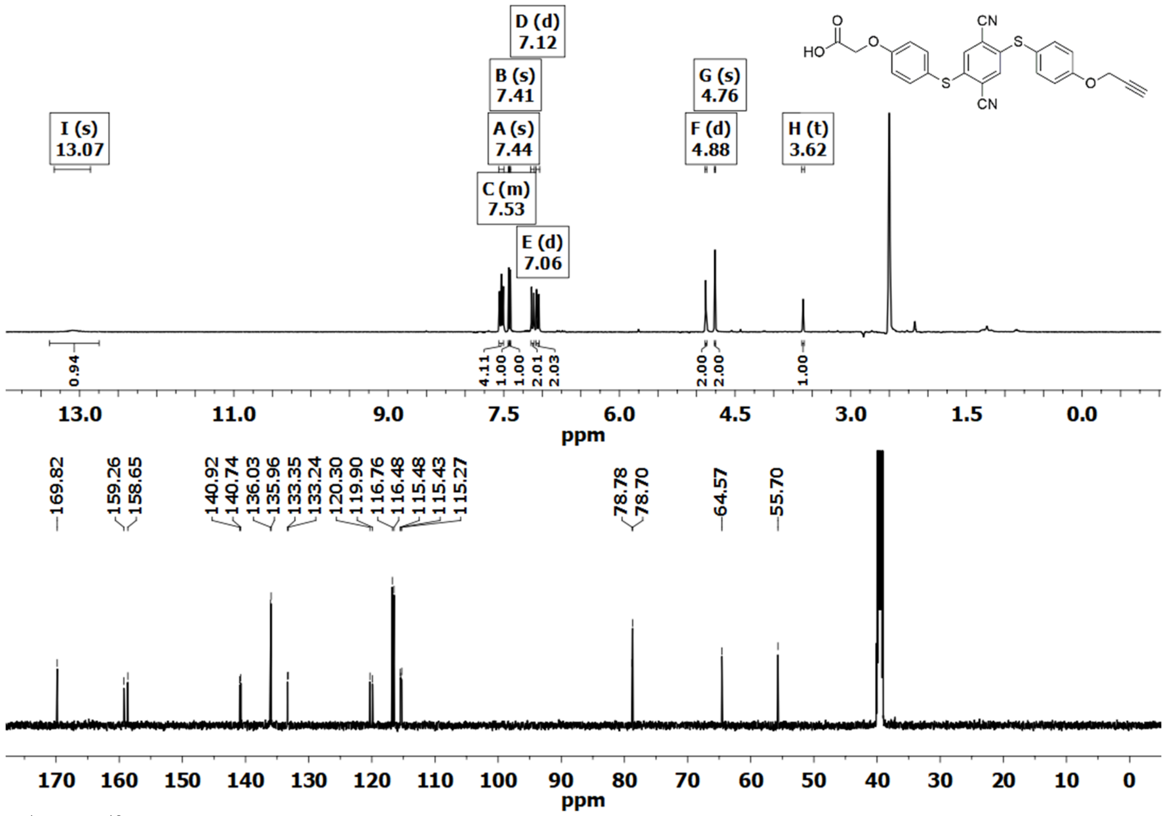

Figure S6: ^{1}H and ^{13}C NMR of compound **(G)**. (DMSO-*d*6)

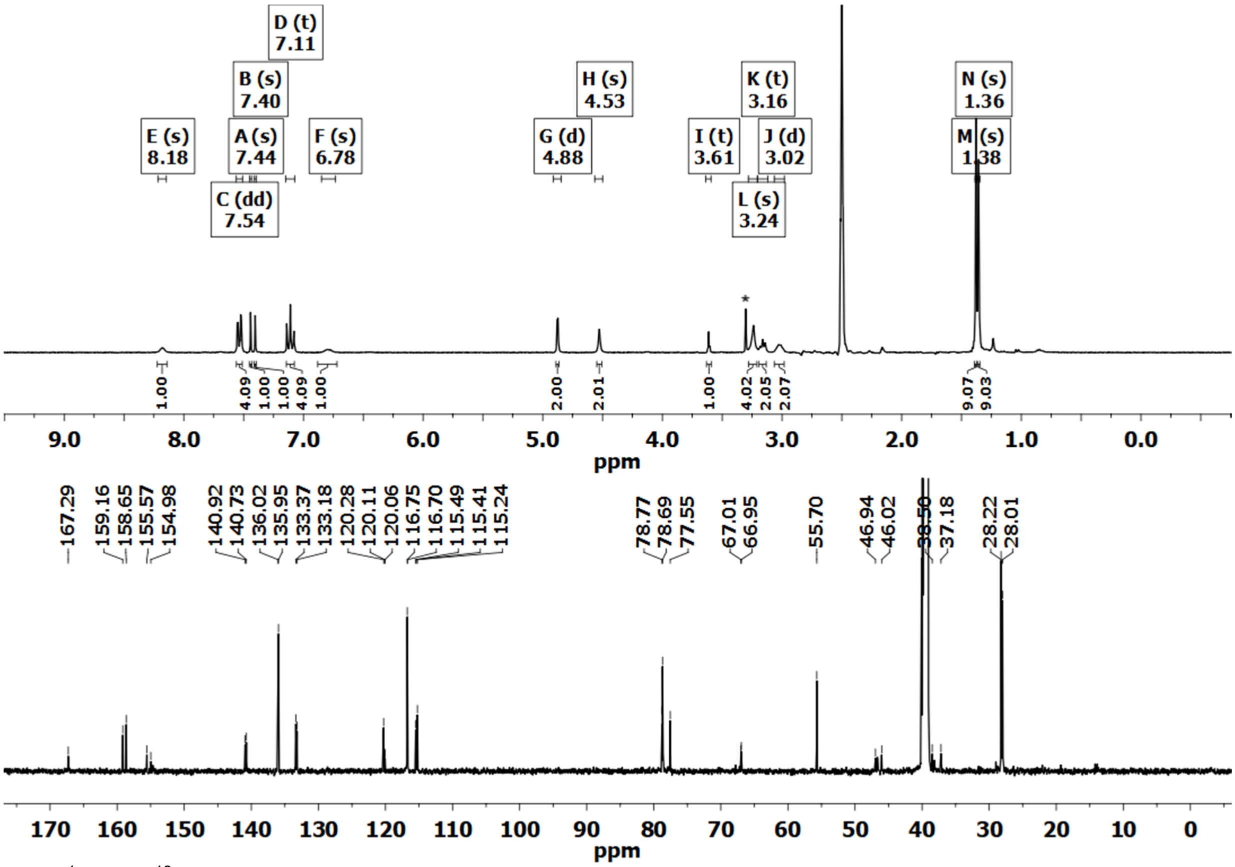

Figure. S7: ^{1}H- and ^{13}C-NMR of compound **(H)**. * = water. (DMSO-d_6)

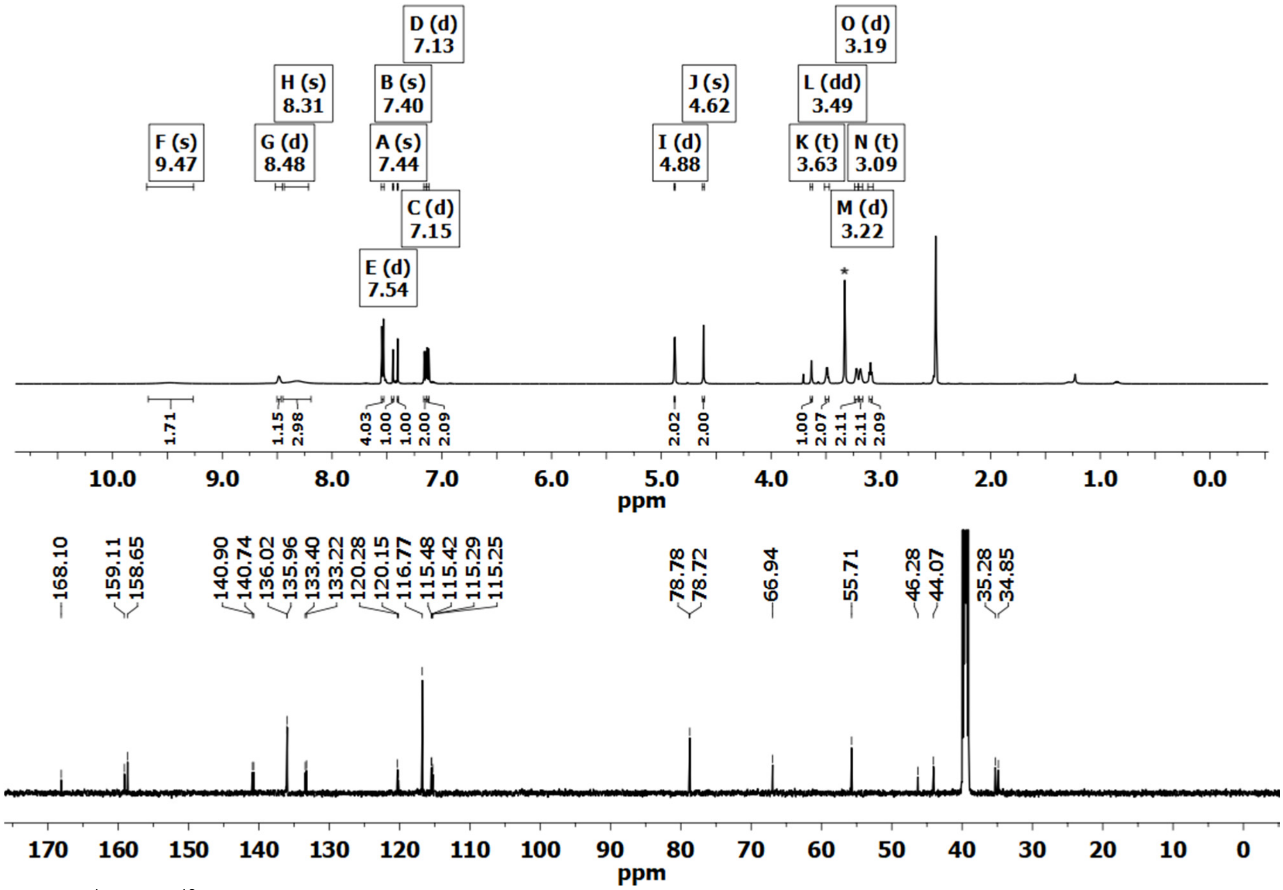

Figure. S8: ^{1}H- and ^{13}C-NMR of compound **(2)**. * = water. (DMSO-d_6)

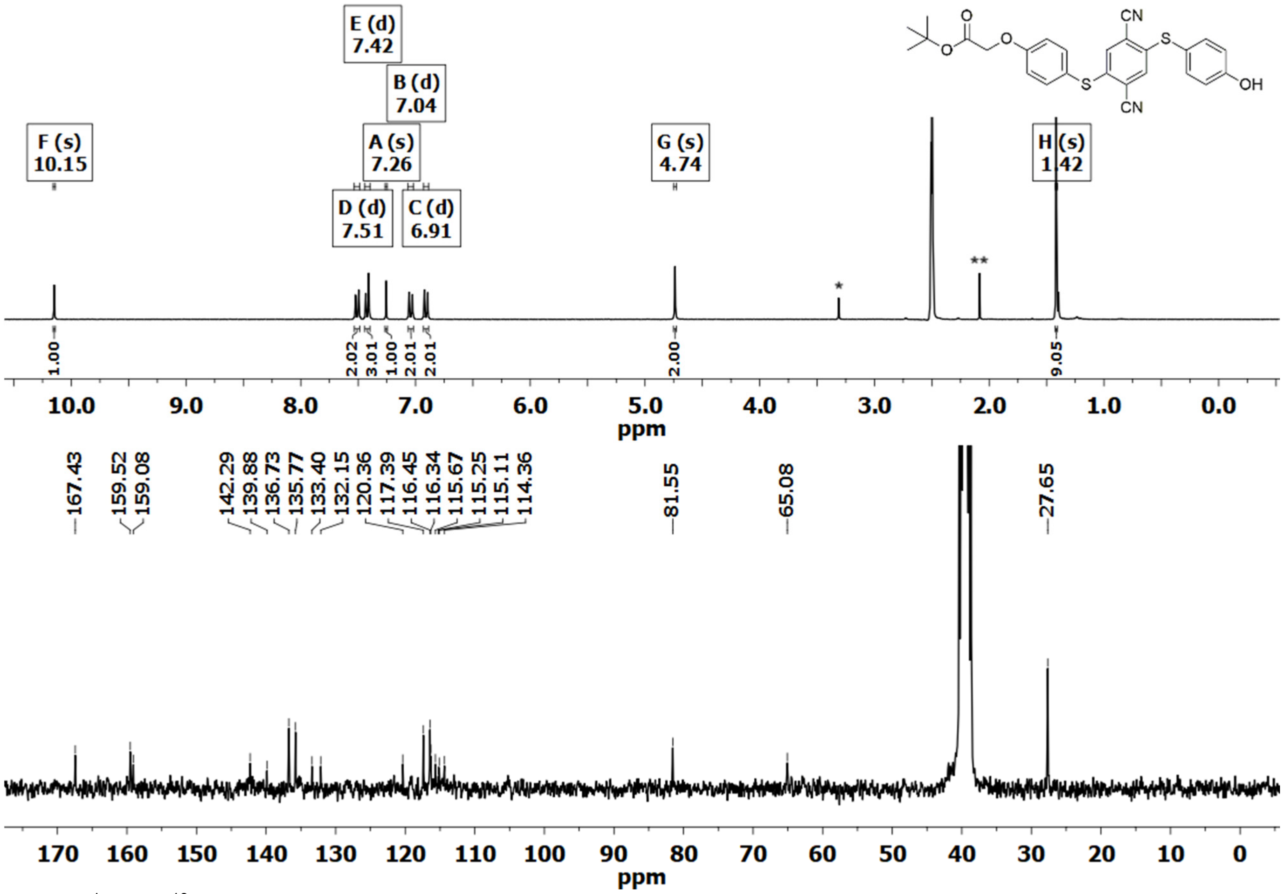

Figure. S9: ^{1}H and ^{13}C NMR of compound **(K)**. * = water, ** = acetone. (DMSO-*d*6)

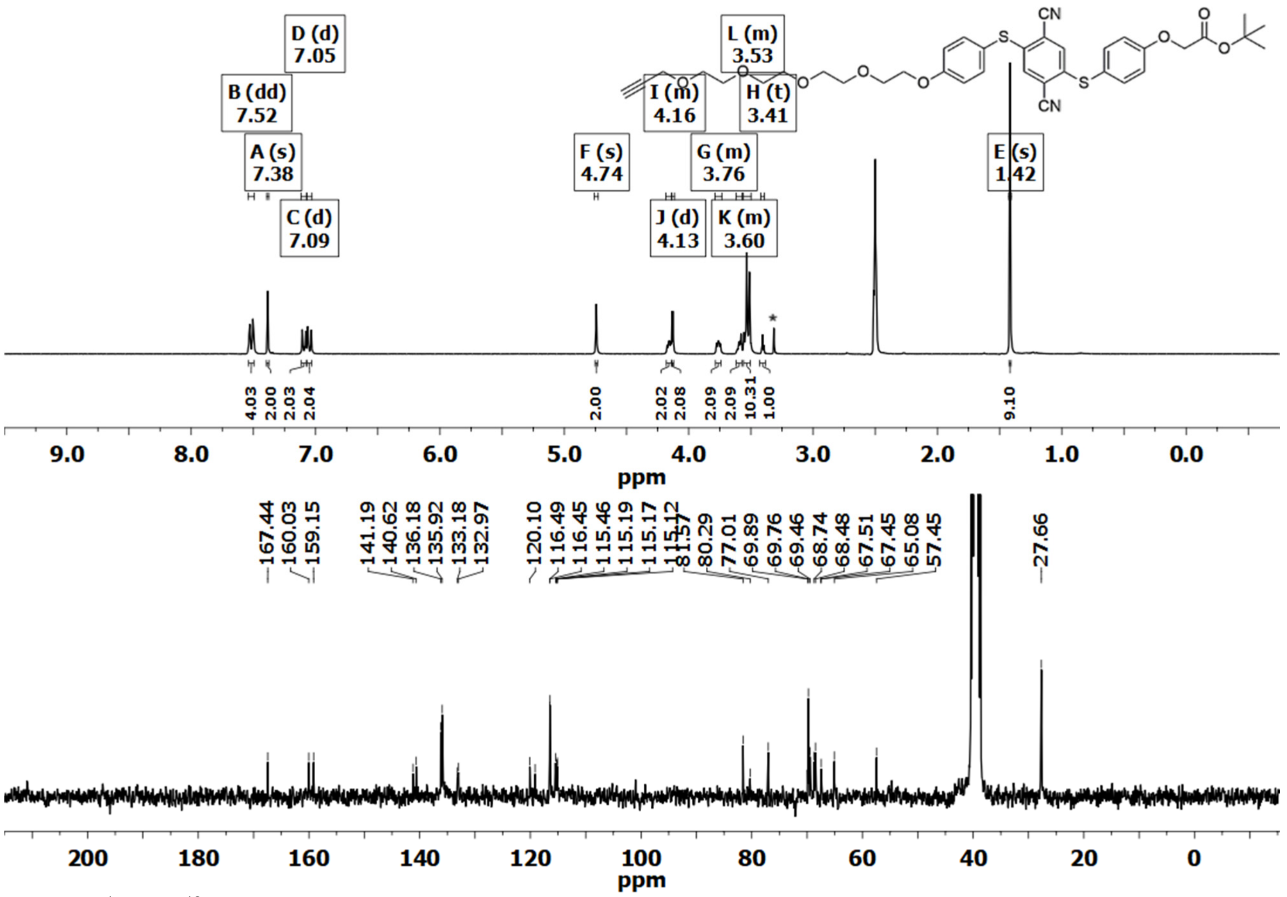

Figure. S10: ^{1}H and ^{13}C NMR of compound **(L)**. * = water. (DMSO-d_6)

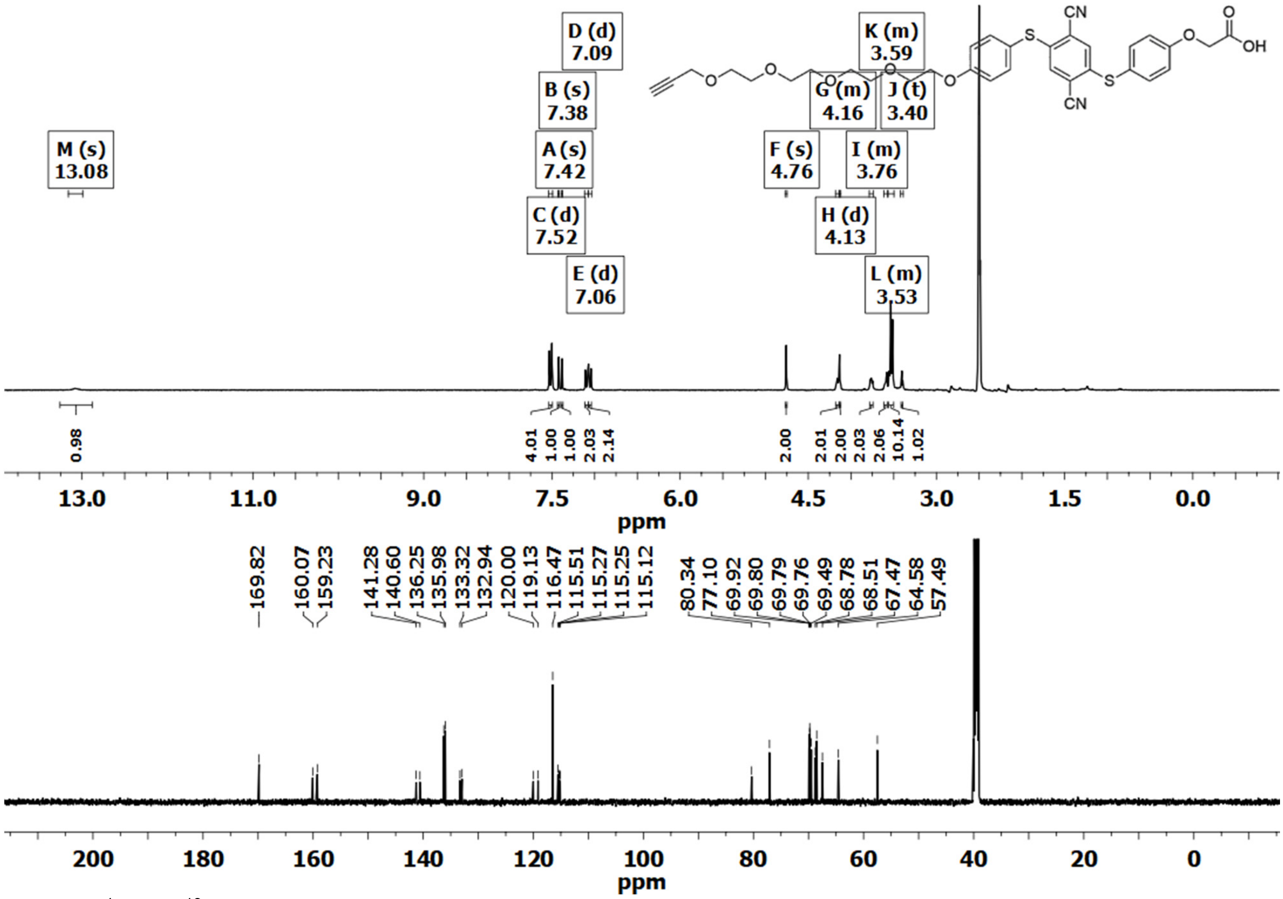

Figure. S11: ^{1}H and ^{13}C NMR of compound **(M)**. (DMSO-d_6)

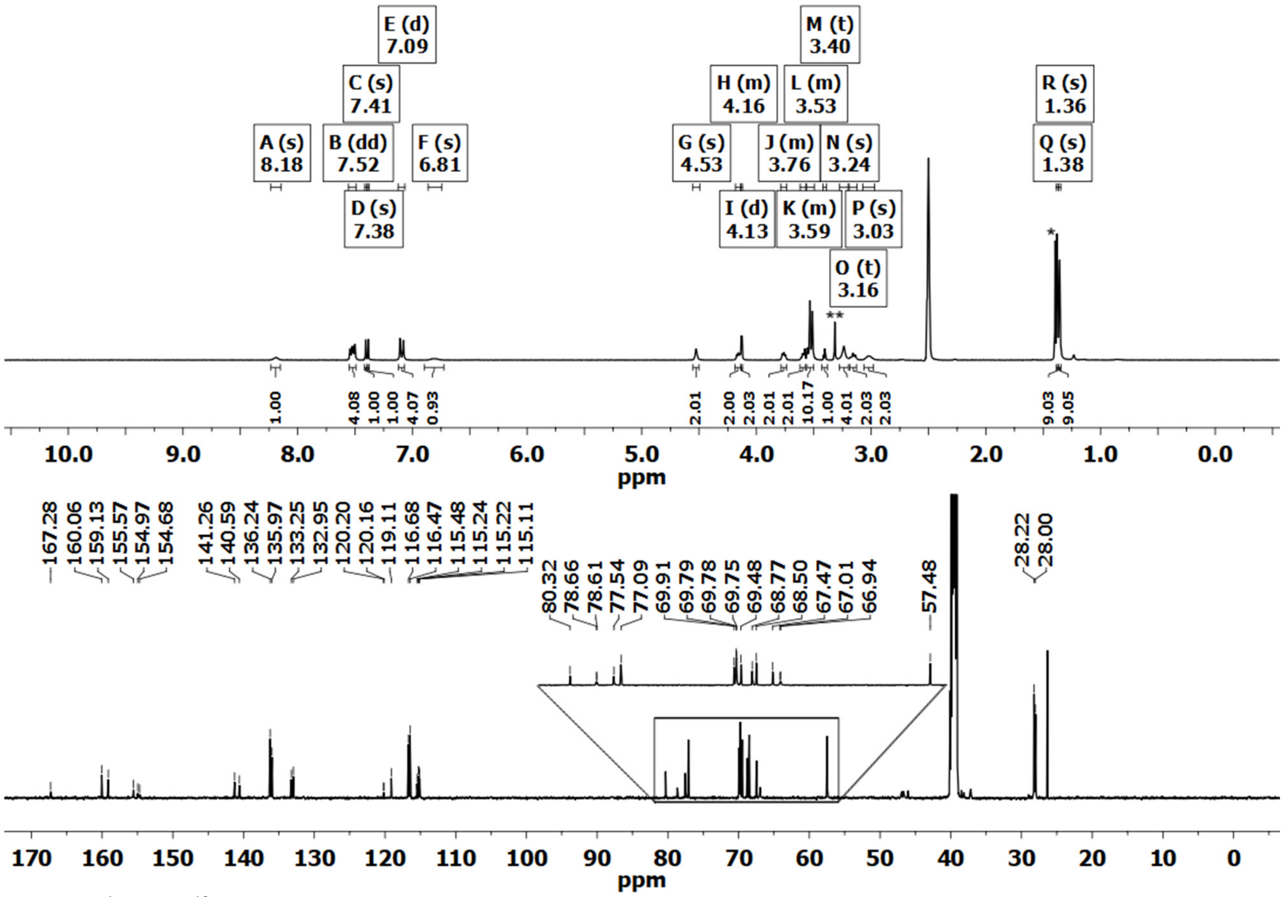

Figure. S12: ^{1}H- and ^{13}C-NMR of compound **(N)**. * = cyclohexane, ** = water. (DMSO-d_6)

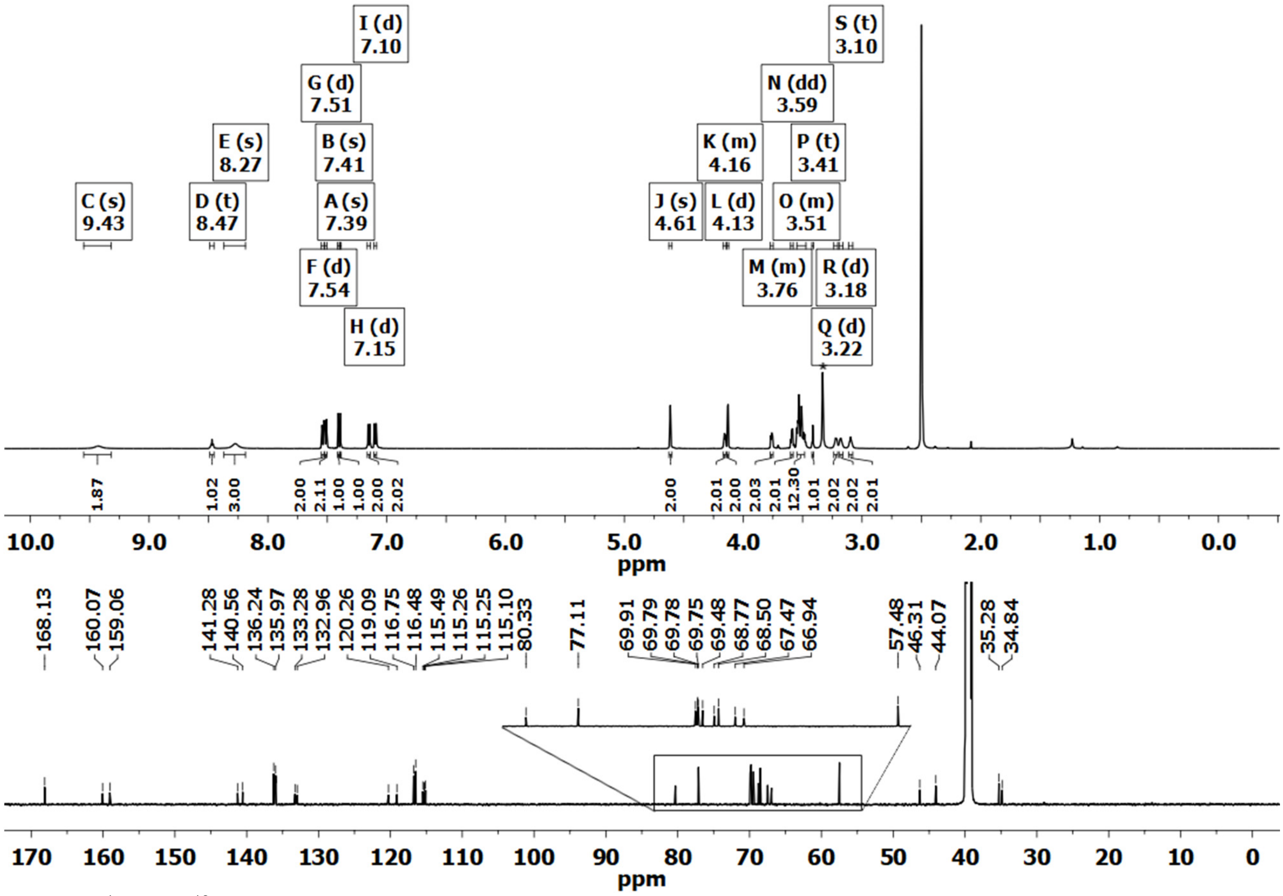

Figure. S13: ^{1}H- and ^{13}C-NMR of compound **(3)**. * = water. (DMSO-d_6)

4. UV/Vis spectroscopy

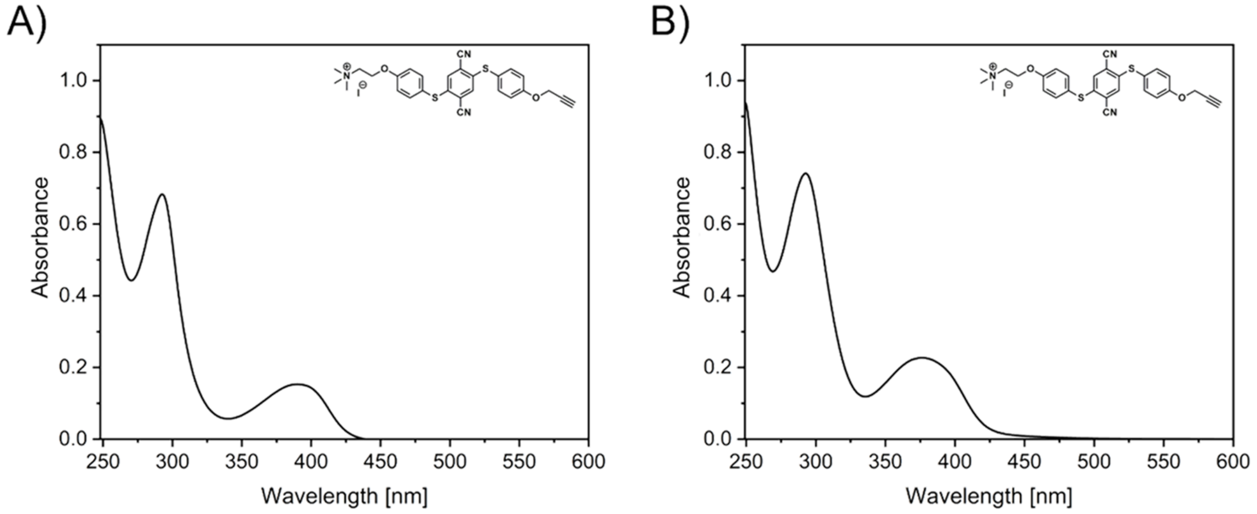

Figure. S14: The absorption spectra in the aggregated state and the molecular structure of **(1)** in A) water/DMF (99.7:0.3) and B) THF/DMF (99.7:0.3). (c = 30 µM)

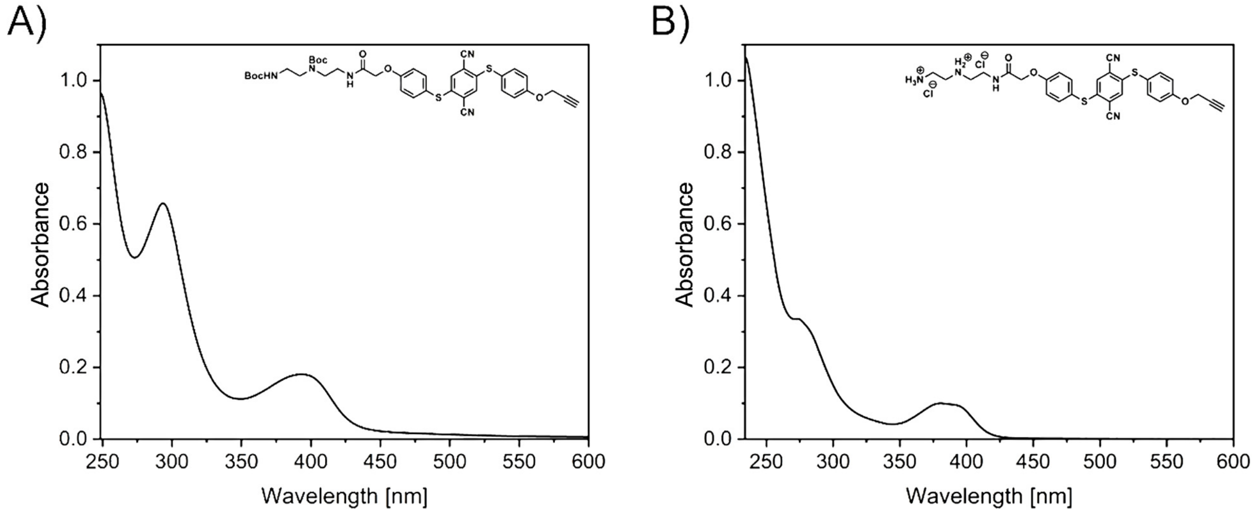

Figure. S15: The absorption spectra in the aggregated state and the molecular structure of A) **(H)** in water/DMF (99.7:0.3) and B) **(2)** in THF/water (99.7:0.3). (c = 30 µM).

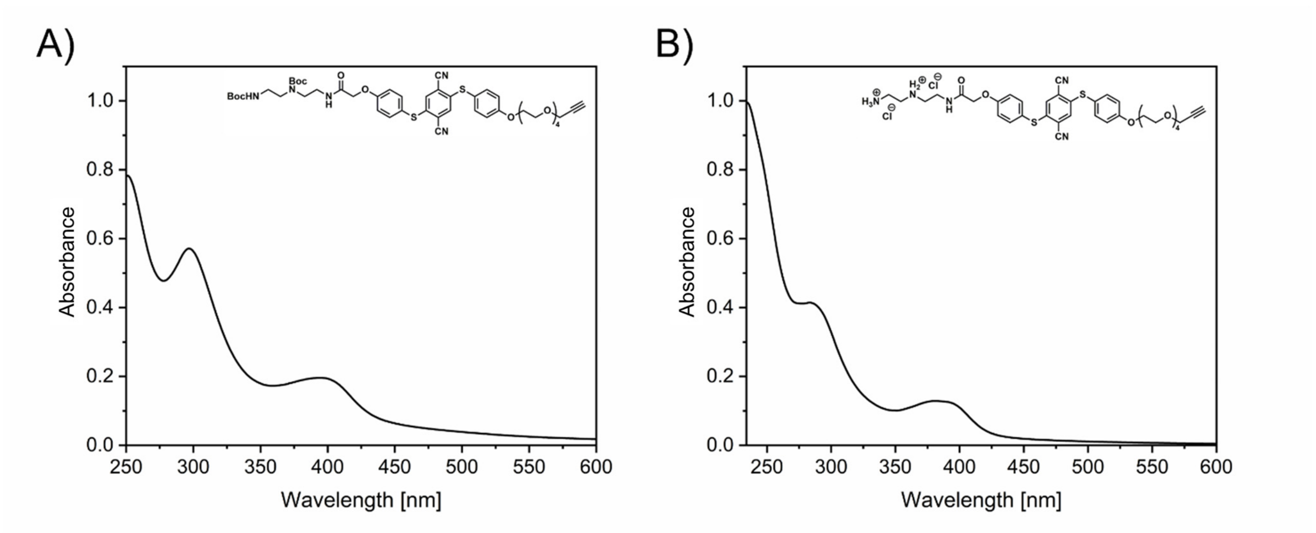

Figure. S16: The absorption spectra in the aggregated state and the molecular structure of A) **(N)** in water/DMF (99.7:0.3) and B) **(3)** in THF/water (99.7:0.3). (c = 30 µM)

5. Fluorescence spectroscopy

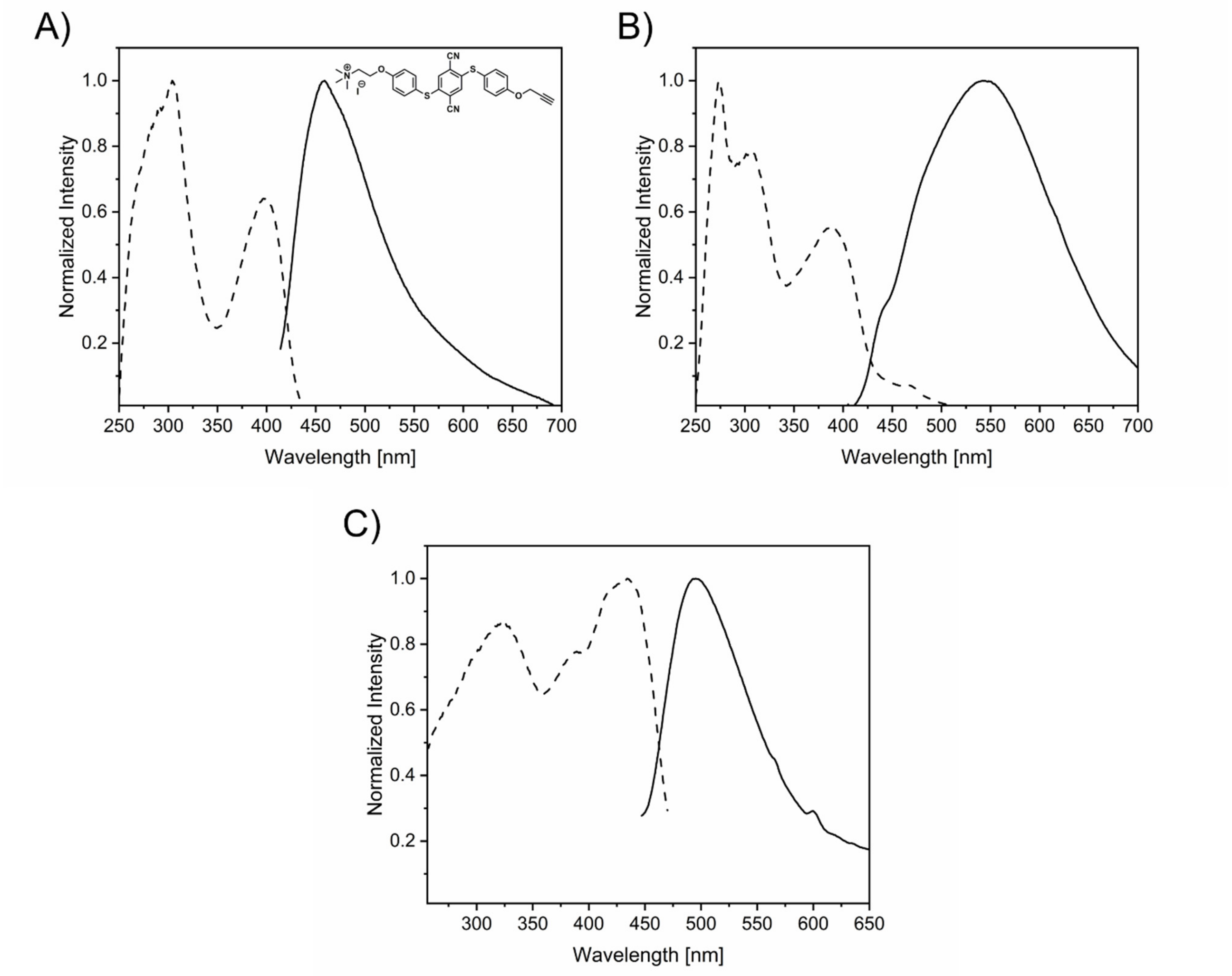

Figure. S17: Normalized excitation and emission spectra of **(1)** in A) the aggregated state in water/DMF (99:1) with the molecular structure, B) the aggregated state in THF/DMF (99:1) and C) the solid state. (c = 100 µM).

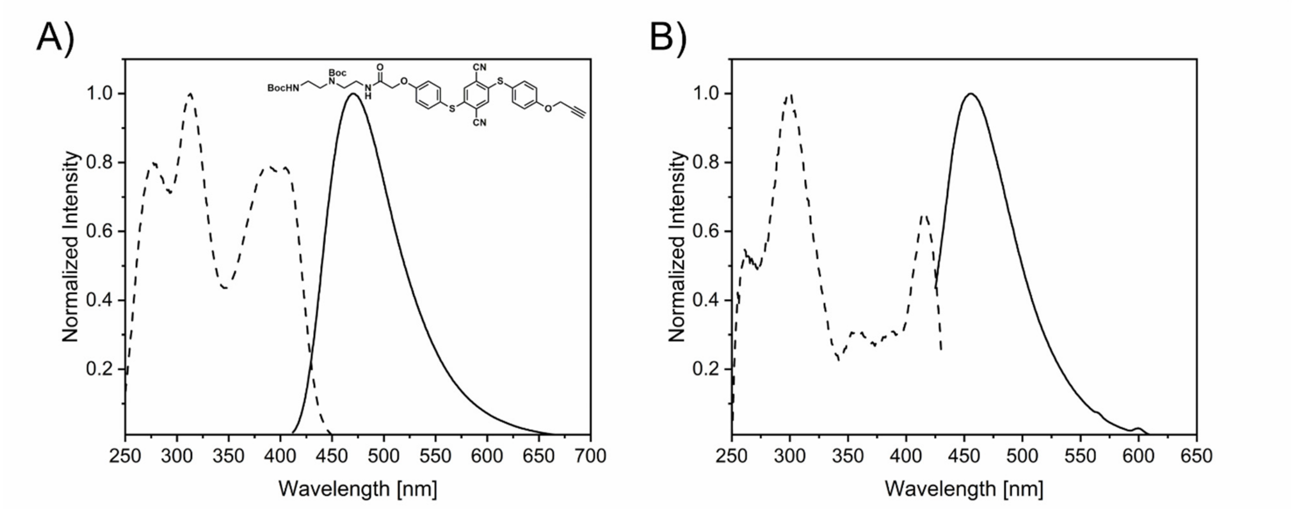

Figure. S18: Normalized excitation and emission spectra of **(H)** in A) the aggregated state in water/DMF (99:1) with the molecular structure and B) the solid state. (c = 100 µM).

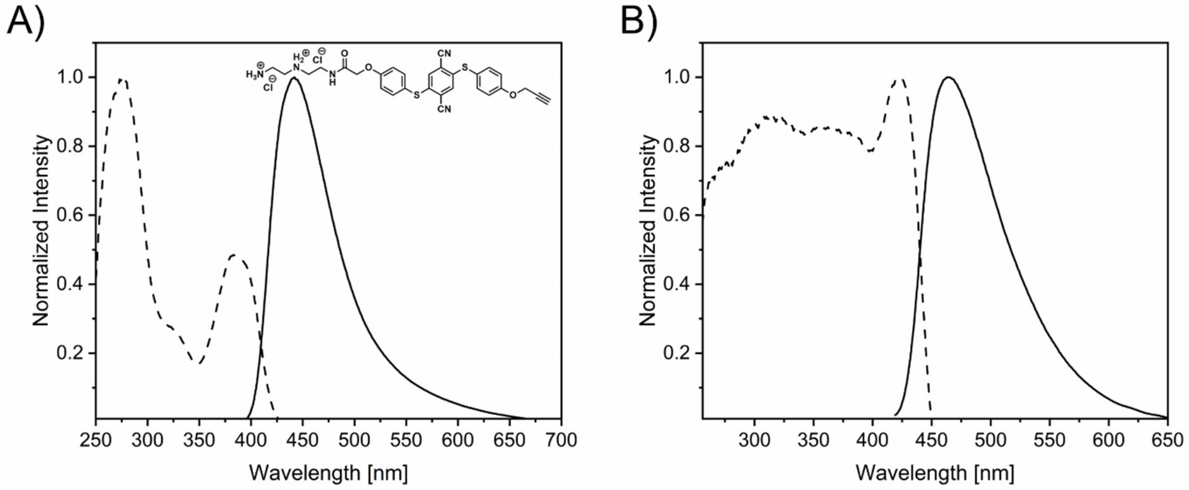

Figure. S19: Normalized excitation and emission spectra of (**2**) in A) the aggregated state in THF/water (99:1) with the molecular structure and B) the solid state. (c = 100 µM).

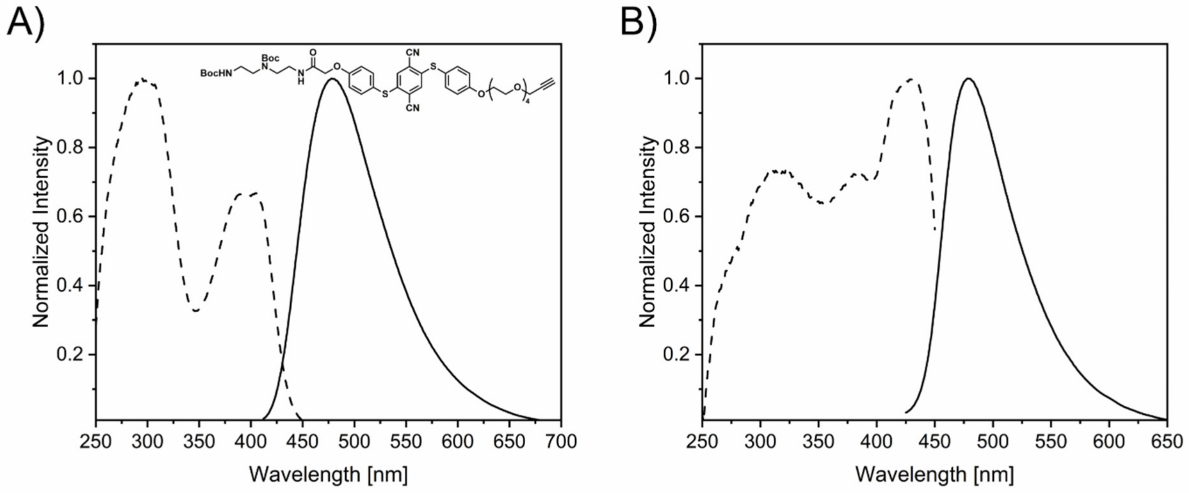

Figure. S20: Normalized excitation and emission spectra of (**N**) in A) the aggregated state in water/DMF (99:1) with the molecular structure and B) the solid state. (c = 100 µM).

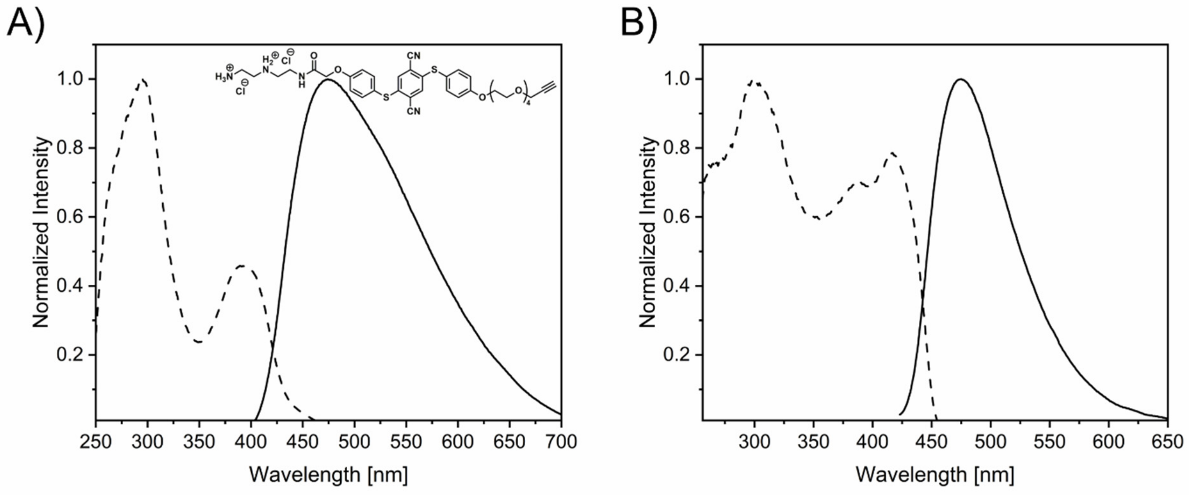

Figure. S21: Normalized excitation and emission spectra of (**3**) in A) the aggregated state in THF/Water (99:1) with the molecular structure and B) the solid state. (c = 100 µM).

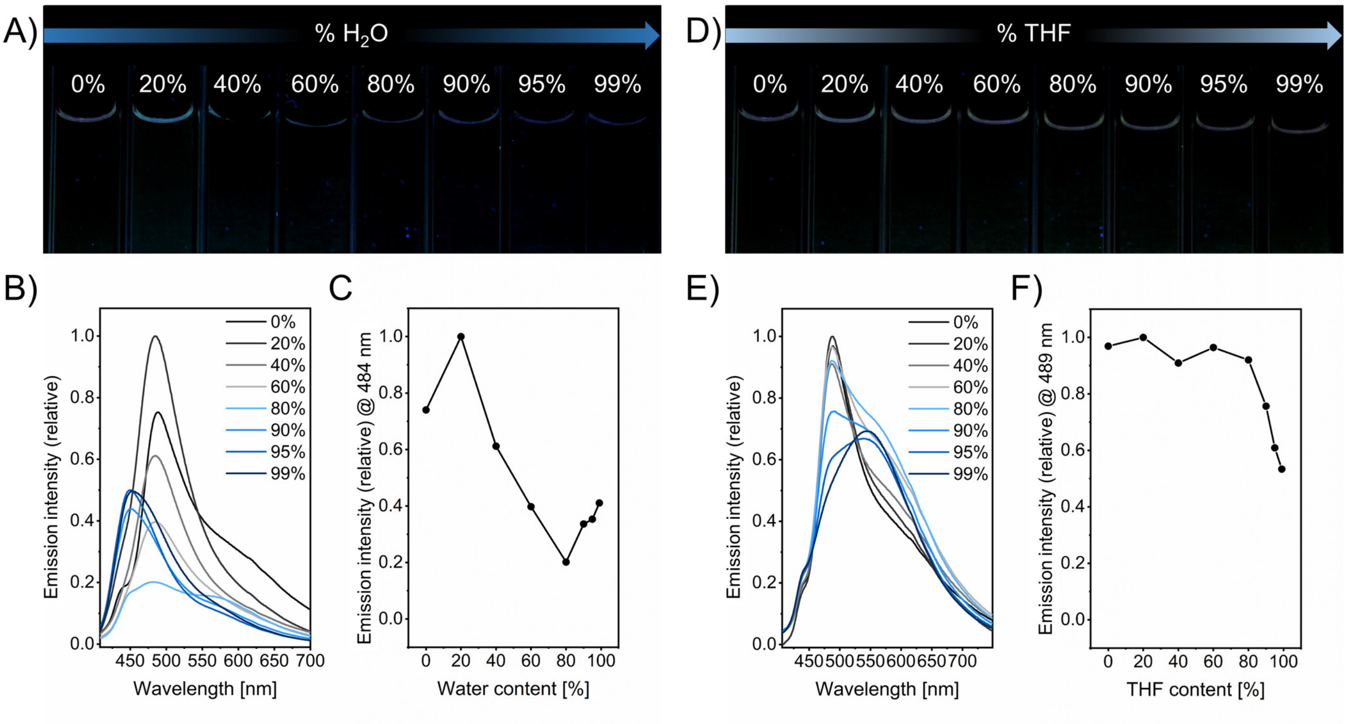

Figure. S22: Solvent dependency of compound (**1**), A) Photographs of cuvettes under UV-light (365 nm) with increasing water content in DMF, B) Emission spectra with varying water content in DMF and C) the emission maximum at 484 nm as a function of the different water content; D) Photographs of cuvettes under UV-light (365 nm) with increasing THF content in DMF, E) Emission spectra with varying THF content in DMF and F) the emission maximum at 489 nm as a function of the different THF content. (c = 100 μM). It is noteworthy that in all cases the emission remains weak.

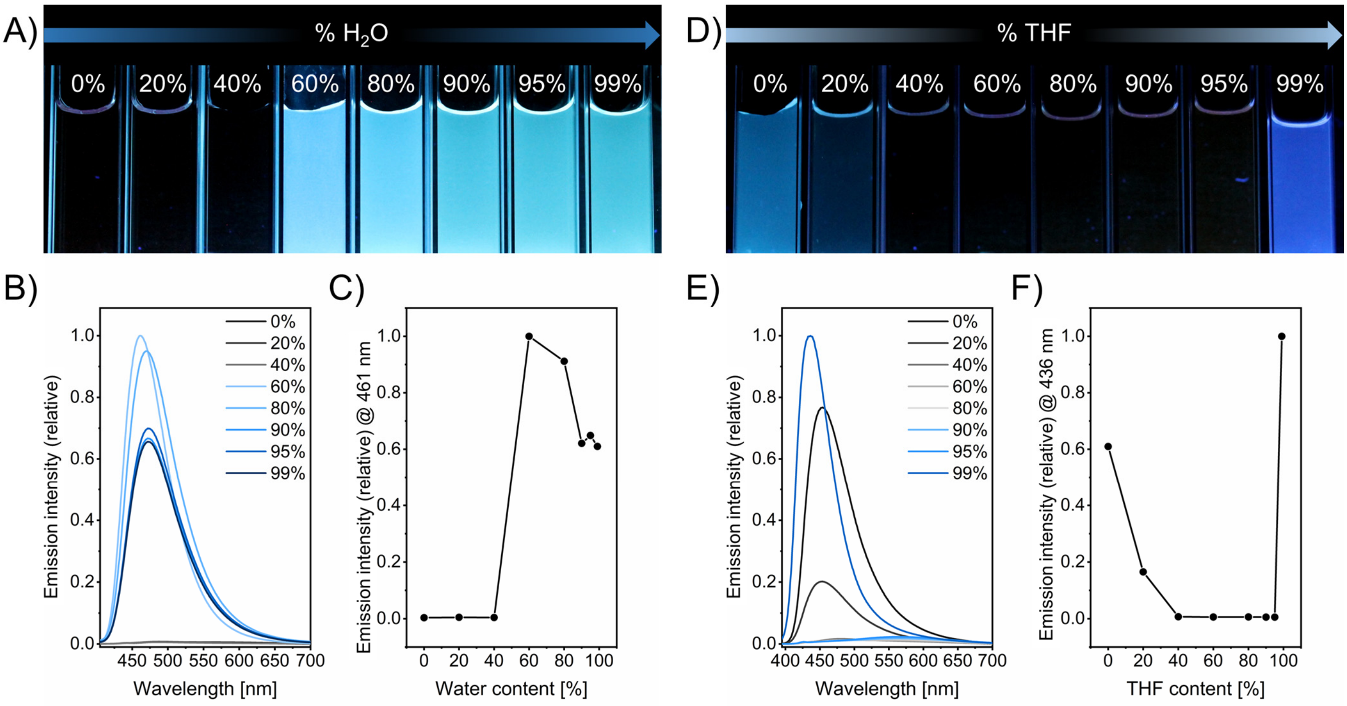

Figure. S23: Solvent dependency of compound (**H**) (left), A) Photographs of cuvettes under UV-light (365 nm) with increasing water content (bad solvent) in DMF (good solvent), B) Emission spectra with varying water content in DMF and C) the emission maximum at 461 nm as a function of the different water content; and (**2**) (right), D) Photographs of cuvettes under UV-light (365

nm) with increasing THF content in water, E) Emission spectra with increasing THF content (bad solvent) in water (good solvent) F) the emission maximum at 436 nm as a function of different THF content. (c = 100 µM).

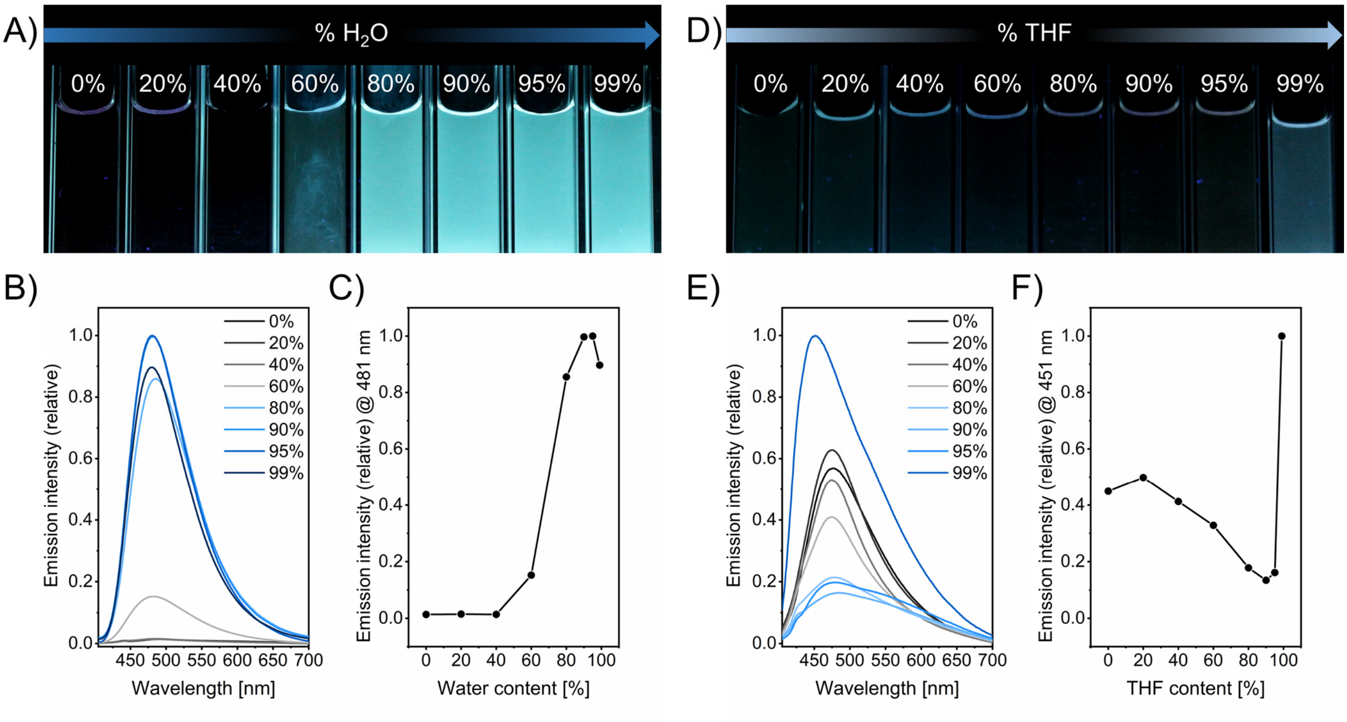

Figure. S24: Solvent dependency of compound (**N**) (left), A) Photographs of cuvettes under UV-light (365 nm) with increasing water content (bad solvent) in DMF (good solvent), B) Emission spectra with varying water content in DMF and C) the emission maximum at 481 nm as a function of the different water content; and (**3**) (right), D) Photographs of cuvettes under UV-light (365 nm) with increasing THF (bad solvent) content in water (good solvent), E) Emission spectra with increasing THF (bad solvent) content in water (good solvent) and F) the emission maximum at 451 nm as a function of the different THF content. (c = 100 µM).

References:

[1] J.-Z. Cheng, C.-C. Lin, P.-T. Chou, A. Chaskar and K.-T. Wong, *Tetrahedron* **2011**, *67*, 734-739.
[2] J. S. A. Ishibashi, J. L. Marshall, A.Mazière, G. J. Lovinger, B. Li, L. N. Zakharov, A. Dargelos, A. Graciaa, A. Chrostowska, S.-Y. Liu, *J. Am. Chem. Soc.* **2014**, *136*, 15414-15421
[3] L. Rojas-Sánchez, V. Sokolova, S. Riebe, J. Voskuhl and M. Epple, *ChemNanoMat*, **2019**, *5*, 436-446.
[4] S. Zhu, J. Zhang, G. Vegesna, F.-T. Luo, S. A. Green and H. Liu, *Org. Lett.* **2011**, *13*, 438-441.
[5] S. Riebe, C. Vallet, F. van der Vight, D. Gonzalez-Abradelo, C. Wölper, C. A. Strassert, G. Jansen, S. Knauer, J. Voskuhl, *Chem. Eur. J,* **2017**, *23*, 13660-13668.
